# Design, Synthesis, and Anti-Leukemic Evaluation of a Series of Dianilinopyrimidines by Regulating the Ras/Raf/MEK/ERK and STAT3/c-Myc Pathways

**DOI:** 10.3390/molecules29071597

**Published:** 2024-04-03

**Authors:** Chaoyan Wang, Bo Wang, Yu Mou, Xiang Liu, Qiqing Chen, Weidong Pu, Qing Rao, Chunlin Wang, Jingrui Song, Yubing Huang, Longjia Yan, Lei Huang, Yanmei Li

**Affiliations:** 1 State Key Laboratory of Functions and Applications of Medicinal Plants, Guizhou Medical University, Guiyang 550014, China; wangchaoyan3696@163.com (C.W.); wangbo9626@126.com (B.W.); mouyiii@163.com (Y.M.); lx00129@126.com (X.L.); chenqiqing07@163.com (Q.C.); puweidong518@163.com (W.P.); raoqing2015@163.com (Q.R.); wangchunlin3323@163.com (C.W.); songjingrui123@snnu.edu.cn (J.S.); huangyubing@gmc.edu.cn (Y.H.); 2 Natural Products Research Center of Guizhou Province, Guiyang 550014, China; 3 College of Pharmacy, Guizhou Medical University, Guiyang 550004, China; 4 College of Basic Medical, Guizhou Medical University, Guiyang 550004, China; 5 School of Pharmaceutical Sciences, Guizhou University, Guiyang 550000, China

**Keywords:** leukemia, apoptosis, Ras/Raf/MER/ERK, STAT3/c-Myc, dianilinopyrimidines

## Abstract

Although the long-term survival rate for leukemia has made significant progress over the years with the development of chemotherapeutics, patients still suffer from relapse, leading to an unsatisfactory outcome. To discover the new effective anti-leukemia compounds, we synthesized a series of dianilinopyrimidines and evaluated the anti-leukemia activities of those compounds by using leukemia cell lines (HEL, Jurkat, and K562). The results showed that the dianilinopyrimidine analog **H-120** predominantly displayed the highest cytotoxic potential in HEL cells. It remarkably induced apoptosis of HEL cells by activating the apoptosis-related proteins (cleaved caspase-3, cleaved caspase-9 and cleaved poly ADP-ribose polymerase (PARP)), increasing apoptosis protein Bad expression, and decreasing the expression of anti-apoptotic proteins (Bcl-2 and Bcl-xL). Furthermore, it induced cell cycle arrest in G2/M; concomitantly, we observed the activation of p53 and a reduction in phosphorylated cell division cycle 25C (p-CDC25C) / Cyclin B1 levels in treated cells. Additionally, the mechanism study revealed that **H-120** decreased these phosphorylated signal transducers and activators of transcription 3, rat sarcoma, phosphorylated cellular RAF proto-oncogene serine / threonine kinase, phosphorylated mitogen-activated protein kinase kinase, phosphorylated extracellular signal-regulated kinase, and cellular myelocytomatosis oncogene (p-STAT3, Ras, p-C-Raf, p-MEK, p-MRK, and c-Myc) protein levels in HEL cells. Using the cytoplasmic and nuclear proteins isolation assay, we found for the first time that **H-120** can inhibit the activation of STAT3 and c-Myc and block STAT3 phosphorylation and dimerization. Moreover, **H-120** treatment effectively inhibited the disease progression of erythroleukemia mice by promoting erythroid differentiation into the maturation of erythrocytes and activating the immune cells. Significantly, **H-120** also improved liver function in erythroleukemia mice. Therefore, **H-120** may be a potential chemotherapeutic drug for leukemia patients.

## 1. Introduction

Acute erythroid leukemia (AEL) is an uncommon subordinate type of acute myeloid leukemia (AML). Its cytology is characterized by a significant accumulation of erythroid cells with arrested maturation [1,2]. Treatment for patients with AEL is generally similar to patients with other types of AML [3]. Currently, chemotherapy combined with cytarabine (AraC) and anthracyclines is still the standard treatment for AML [4], including AEL [5]. However, there are no prospective clinical trials for AEL patients [6] and no proven effective treatment strategies for AEL [7,8]. To improve outcomes for patients with AEL, a concerted focus on research and treatment development for AEL is necessary [9].

The up-regulation of the tumor suppressor p53 has been demonstrated to be significant in leukemia [10]. In response to DNA damage, hypoxia, oncogene activation, and various other stimuli, p53 undergoes up-regulation, leading to diverse cellular responses such as growth arrest, apoptosis, and DNA repair [11,12]. These responses are orchestrated by multiple factors, among which CDC25C plays a crucial role. CDC25C is a target of the p53 tumor suppressor, and its expression is modulated by p53, either being induced or repressed. It has been established that CDC25C is necessary for G2/M arrest [10]. Uncontrolled leukemia proliferation and reduced sensitivity to apoptotic-inducing agents are typically associated with the activation of pro-survival pathways [11]. The Ras/Raf/MEK/ERK pathway regulates the activity of many proteins involved in apoptosis [12]. Due to its involvement in sensitivity to leukemia therapy, this pathway is frequently implicated in the field. Many of the effects of the pathway on apoptosis are mediated by ERK phosphorylation of key apoptotic effector molecules [13]. ERK phosphorylates transcription factors that influence the transcription of the Bcl-2 family in the regulation of apoptosis. Increased expression of Bcl-2 and Bcl-xL is observed in some leukemias [11]. Regulating the expression of Bcl-2 and Bcl-xL could improve leukemia therapy and ameliorate human health. As a signal transducer, STAT3 can affect proliferation, differentiation, and apoptosis in cancer cells [14]. Structural activation of STAT3 has been shown in many forms of leukemia, including erythroleukemia [15,16]. Clinical correlations show that constitutively active STAT3 in patients indicates poor prognosis, in addition to more severe anemia, more proliferation of blasts in the blood, and promoting resistance to chemotherapy [17]. Inhibition of abnormal activation of STAT3 has been proven to be effective in the treatment of leukemia [18]. Molecular studies indicate that STAT3 mediates transcriptional activation of the transcription factor c-Myc directly [16]. c-Myc plays a crucial role in the terminal differentiation of hematopoietic cells via regulating the transcription of downstream target genes [19]. A study found that several vital oncogenes in acute myeloid leukemia (AML), such as AML1-ETO, PML/RARa, and PLZF/RARa, induce leukemogenesis by activating c-Myc [20,21]. The importance of c-Myc in myeloid leukemogenesis is further demonstrated by the induction of myeloid leukemia using ectopically expressed c-Myc in murine bone marrow (BM) progenitors [20].

Small molecules are highly favored in the development of anti-tumor drugs and the discovery of lead compounds because of their unique chemical and biological properties [22]. **H-120** is a derivative of *N*-Phenyl-2-pyrimidinamine. In this study, we evaluated the effect of **H-120** on HEL cells and its therapeutic effect in erythroleukemia mice. The results showed that **H-120** may achieve an anti-erythroleukemia effect through the Ras/Raf/MEK/ERK signal pathway and STAT3/c-Myc pathway in vitro, and it has a good effect on the treatment of erythroleukemia mice, suggesting that **H-120** may be a potential chemotherapeutic drug for patients with erythroleukemia.

## 2. Results

### 2.1. Chemistry

**Scheme 1:**
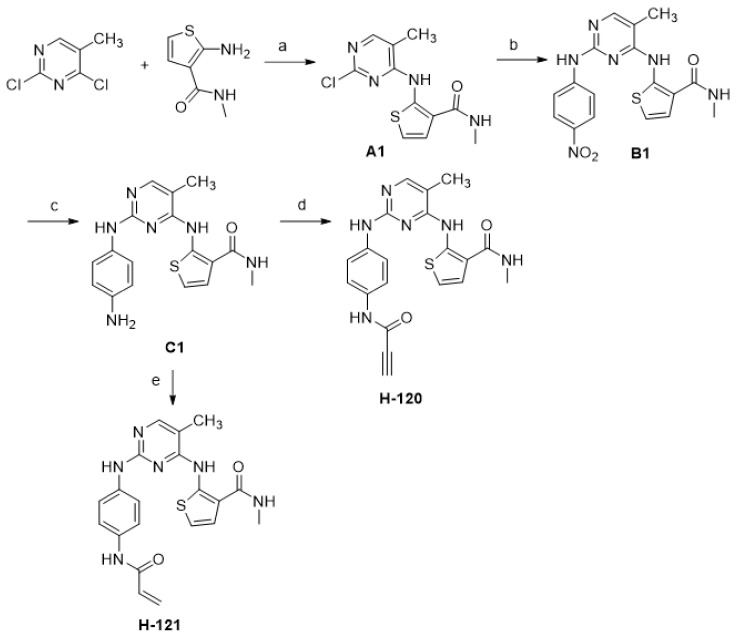
Reagents and conditions: (**a**) EtOH, NaHCO_3_, 70 °C, 12 h; (**b**) TFE, 4-nitroaniline /TFA, N_2_, 70 °C, 12 h; (**c**) MeOH, Pd/C, H_2_, rt, 24 h; (**d**) DMF, DIEA, propiolic acid, HATU, rt, 24 h; (**e**) DCM, DCM, DIEA, acryloyl chloride, rt, 24 h.

The compound **H-120** is depicted in Scheme 1. Initially, 2,4-dichloro-5-methylpyrimidine reacted with 2-amino-*N*-methylthiophene-3-formamide to produce compound **A1** with a 54% yield. Subsequently, the 2-chloro group in the pyrimidine ring was substituted by 4-nitroaniline to give compound **B1** with a 41% yield. The nitro group of **B1** was reduced to an amino group in the presence of Pd/C, and compound **C1** was obtained with a 72% yield. At last, compound **C1** reacted with propiolic acid, and we obtained the compound **H-120** in a 48% yield.

The 2,4-dichloropyrimidine is often used as an intermediate compound for the synthesis of various drugs. Its 4-position chlorine atom is more prone to nucleophilic substitution than the 2-position chlorine atom. In this study, compound **A1** was synthesized through the nucleophilic substitution reaction between 2,4-dichloro-5-methylpyrimidine and 2-amino-*N*-methylthiophene-3-carboxamide. Then, the structures of **A1** were numbered, and each signal in the ^1^H-NMR and ^13^C-NMR spectra was assigned the corresponding proton and carbon number (Supplementary Figure S2). Additionally, the NOESY spectra of compound **A1** revealed a correlation between the C4-N-proton and the C5 (CH_3_)-carbon (pyrimidine ring) (Supplementary Figure S7), suggesting a substitution at the 4-position of 2,4-dichloro-5-methylpyrimidine.

**Scheme 2:**
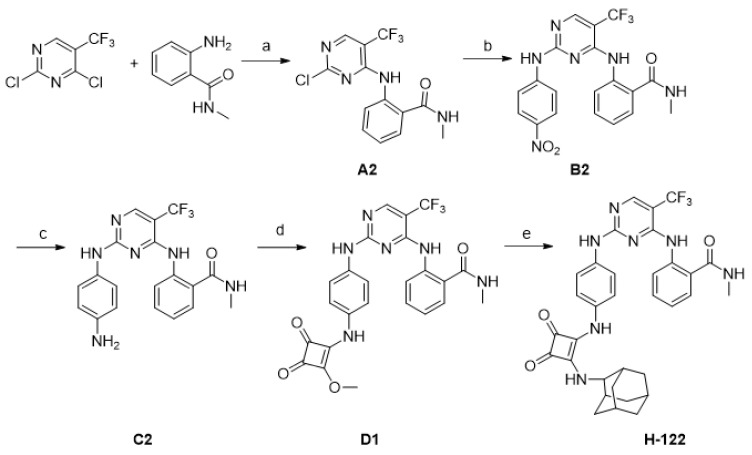
Reagents and conditions: (**a**) EtOH, 2-amino-*N*-methylbenzamide, NaHCO_3_, rt, 12 h; (**b**) TFE, 4-nitroaniline, TFA, N_2_, 70 °C, 12 h; (**c**) MeOH, Pd/C, H_2_, rt, 24 h; (**d**) DMF, dimethyl squarate, DIEA, rt, 12 h; (**e**) DMF, (1*R*,3*R*,5*S*)-adamantan-1-amine, DIEA, rt, 8 h.

The compounds **H-122** are depicted in Scheme 2. Initially, 2,4-dichloro-5-trifluoromethylpyrimidine reacted with 2-amino-N-methylbenzamide to produce **A2** with a 55% yield. Subsequently, the 2-chloro group in the pyrimidine ring was substituted by 4-nitroaniline to give **B2** with a 49% yield. The nitro group of **B2** was reduced to an amino group in the presence of Pd/C, and **C2** was obtained with a 76% yield. Then, **C2** successfully reacted with dimethyl squarate to obtain intermediate **D1** in a 62% yield. Finally, **D1** reacted with (1*R*,3*R*,5*S*)-adamantan-1-amine, giving the compound **H-122** in a 39% yield.

**Scheme 3:**
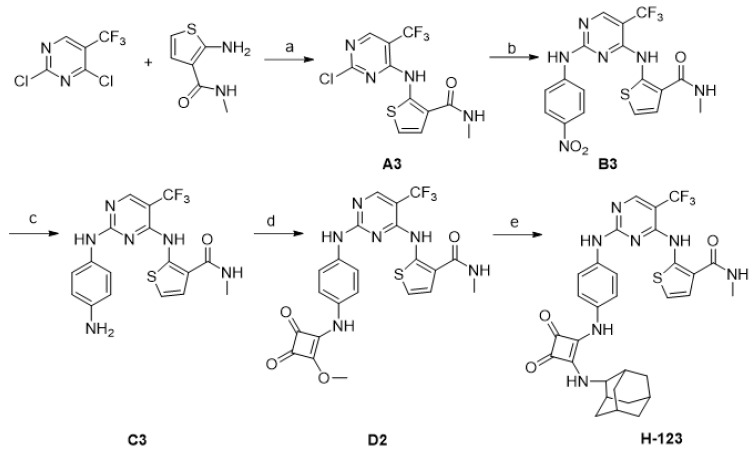
Reagents and conditions: (**a**) EtOH, NaHCO_3_, 70 °C, 12 h; (**b**) TFE, 4-nitroaniline, TFA, 70 °C, N_2_, 12 h; (**c**) MeOH, Pd/C, H_2_, 24 h; (**d**) DMF, dimethyl squarate, DIEA, rt, 12 h; (**e**) DMF, (1*R*,3*R*,5*S*)-adamantan-1-amine, DIEA, rt, 8 h.

The compounds **H-123** are depicted in Scheme 3. Initially, 2,4-dichloro-5-trifluoromethylpyrimidine reacted with 2-amino-N-methylthiophene-3-carboxamide to produce compound **A3** with a 40% yield. Subsequently, the 2-chloro group in the pyrimidine ring was substituted by 4-nitroaniline to give compound **B3** with a 43% yield. The nitro group of **B3** was reduced to amino group in the presence of Pd/C, and compound **C3** was obtained with a 35% yield. Then, **C3** successfully reacted with dimethyl squarate to obtain intermediate **D2** in a 57% yield. Finally, **D2** reacted with (1*R*,3*R*,5*S*)-adamantan-1-amine, giving the compound **H-123** in a 37% yield.

### 2.2. Anti-Leukemia Activities of N-Phenyl-2-Pyrimidinamine Derivatives

**Figure 1:**
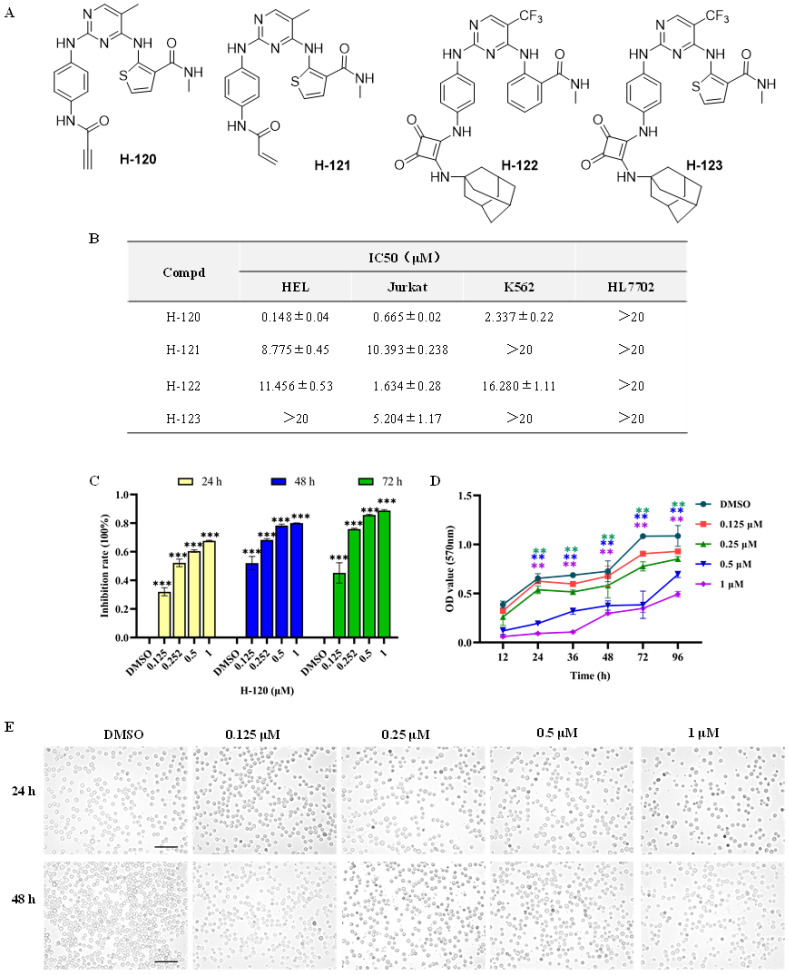
**H-120** inhibited the proliferation of HEL cells. (**A**) Dianilinopyrimidine derivatives. (**B**) cytotoxicity (IC50) of dianilinopyrimidines against indicated cell lines. (**C**) HEL cells treated with various concentrations of **H-120** were analyzed for cell viability by MTT assay. (**D**) HEL cells were incubated with **H-120** for different times, then the growth curve was drawn using GraphPad Prism 8. (**E**) The morphology of HEL cells was observed and photographed using an inverted microscope (magnification, ×200; scale bar = 100 μm). Data are represented as mean ± SD (n = 3; ** *p* < 0.01, *** *p* < 0.001, vs. control).

To estimate the effects of *N*-Phenyl-2-pyrimidinamine derivatives (Figure 1A) on HEL, K562, and Jurkat cells, we treated these cells with the compounds at different concentrations (1–20 μM) for 48 h. The MTT assay results show that **H-120** and **H-122** could selectively inhibit the proliferation of HEL, K562, and Jurkat cells. **H-121** could selectively impair the proliferation of HEL and Jurkat cells. **H-123** blocked the proliferation of the Jurkat cells. Those four compounds had no toxicity to the HL7702 cell line. Moreover, **H-120** was the most effective on HEL cells, and the IC50 was 0.148 ± 0.04 μM (Figure 1B).

### 2.3. H-120 Suppressed HEL Cell Proliferation

To validate the effects of **H-120** on the viability of HEL cells, we treated the cells with the compound at different concentrations (1–20 μM) for 24 h, 48 h, and 72 h. The MTT assay results showed that the **H-120** prevented the HEL cell proliferation in a dose- and time-dependent manner (Figure 1C,D). However, **H-120** was not cytotoxic on normal HL7702 (IC50 > 20 μM) (Figure 1B). The morphology of HEL cells treated with **H-120** gradually crumpled and appeared fragmented (Figure 1E). These data suggest that **H-120** significantly prevented the growth of HEL cells.

### 2.4. H-120 Induces Mitochondrial Damage and Apoptosis in HEL Cells

**Figure 2:**
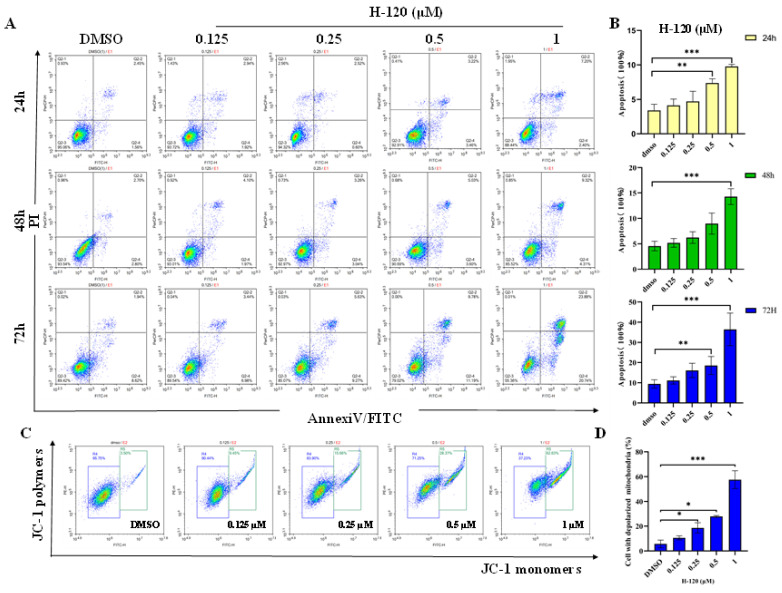

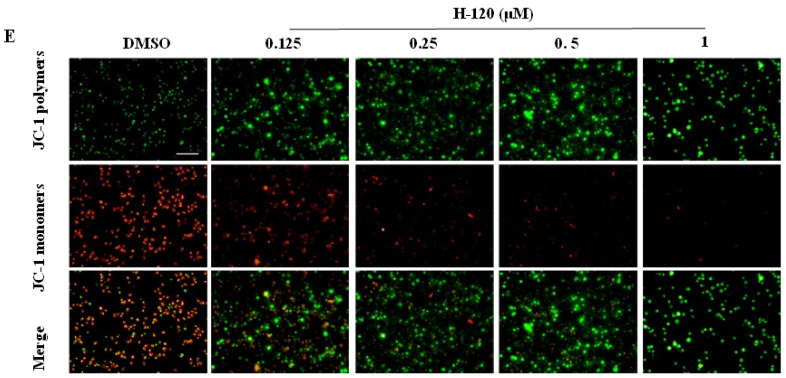
**H-120** induced apoptosis and mitochondrial damage in HEL cells. (**A**) HEL cell apoptosis was detected by annexin-V/propidium iodide double staining using flow cytometry. (**B**) Densitometry of the apoptotic cell percentage at an indicated time interval of **H-120** incubation. (**C**) HEL cells treated with **H-120** for 48 h were stained with JC-1 and analyzed using flow cytometry. (**D**) Graph showing the percentage of depolarized cells in mitochondria at various concentrations of **H-120** treatment. (**E**) Photomicrograph of JC-1 monomers and aggregates after **H-120** incubation at 48 h (magnification, ×200; scale bar = 100 μm). Data are represented as mean ± SD (n = 3; * *p* < 0.05; ** *p* < 0.001; *** *p* < 0.001; vs. the DMSO group).

Apoptosis plays a crucial role in impairing cancer cell viability. To determine whether the **H-120** inhibiting HEL cell growth is caused by apoptosis, we applied HEL cells with different concentrations of **H-120**. The results indicated that the apoptosis rate of HEL cells treated with **H-120** (1 μM) for 48 h was 14.28%, and the apoptosis rate increased to 36.45% for 72 h (Figure 2A,B). To explore the mechanism of apoptosis, we used JC-1 staining to detect the effect of **H-120** on mitochondrial membrane potential. We found that the relative value of the JC-1 monomer was significantly increased with the **H-120** concentration accumulation according to flow cytometry analysis (Figure 2C,D). In addition, the number of JC-1 monomeric cells (green / fluorescence) gradually increased, and the red fluorescence gradually decreased with the concentration increasing (Figure 2E). The results confirmed that **H-120** can induce HEL cell apoptosis by impairing mitochondria.

### 2.5. H-120 Leads to Cell Cycle Arrest and DNA Damage in HEL Cells

**Figure 3:**
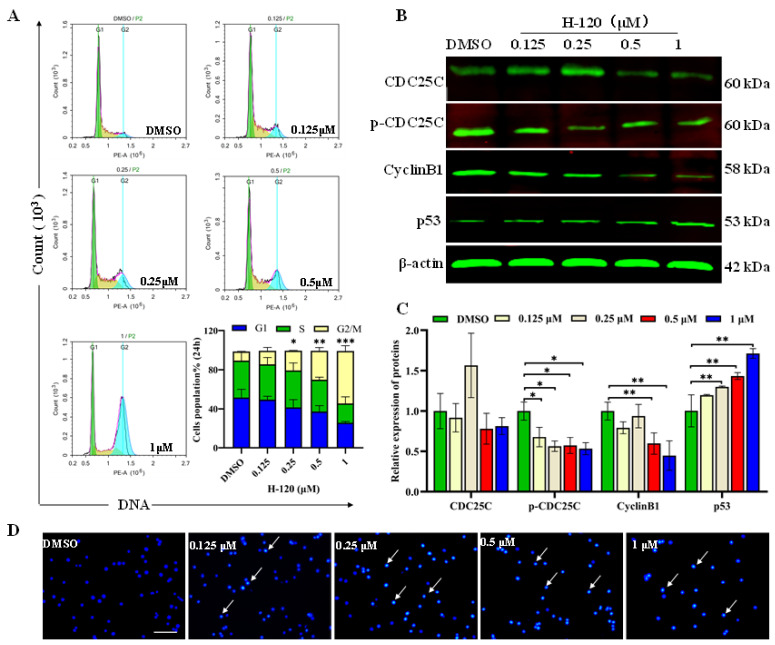
H-120 induces DNA damage in HEL cells and arrest in G2/M phase. (**A**) Cell cycle arrest evaluation by flow cytometry after 24 h, and densitometry plots depicting the variations in the stages of cell cycle arrest. (**B**) The expression of CDC25C, p-CDC25C, CyclinB1, and p53 was measured by Western blotting. (**C**) Quantification of relative protein expression levels of CDC25C, p-CDC25C, CyclinB1, and p53, with β-actin used as loading control. (**D**) Photomicrograph of DNA damage (the pointing of the arrow) at indicated concentrations of **H-120** treatment (magnification, ×200; scale bar = 100 μm). Data are represented as mean ± SD (n = 3, * *p* < 0.05, ** *p* < 0.01, *** *p* <0.001, vs. the DMSO group).

Cell cycle regulation is critical for cell differentiation and proliferation [23]. To investigate whether **H-120** affects the cell cycle of HEL, we treated HEL cells with various concentrations of **H-120**. Flow cytometry results indicated that **H-120** increased the proportion of the G2/M phase in HEL cells in a dose-dependent manner (Figure 3A), indicating that HEL cells were arrested in the G2/M phase. Furthermore, **H-120** up-regulated p53 and decreased the levels of cell cycle proteins p-CDC25C and CyclinB1 in a dose-dependent manner (Figure 3B,C). The Hoechst staining showed that **H-120** caused DNA damage in a dose-dependent manner (Figure 3D), elucidating the apoptotic effect of the **H-120**.

### 2.6. The Effect of H-120 on Apoptosis Proteins in Mitochondrial

**Figure 4:**
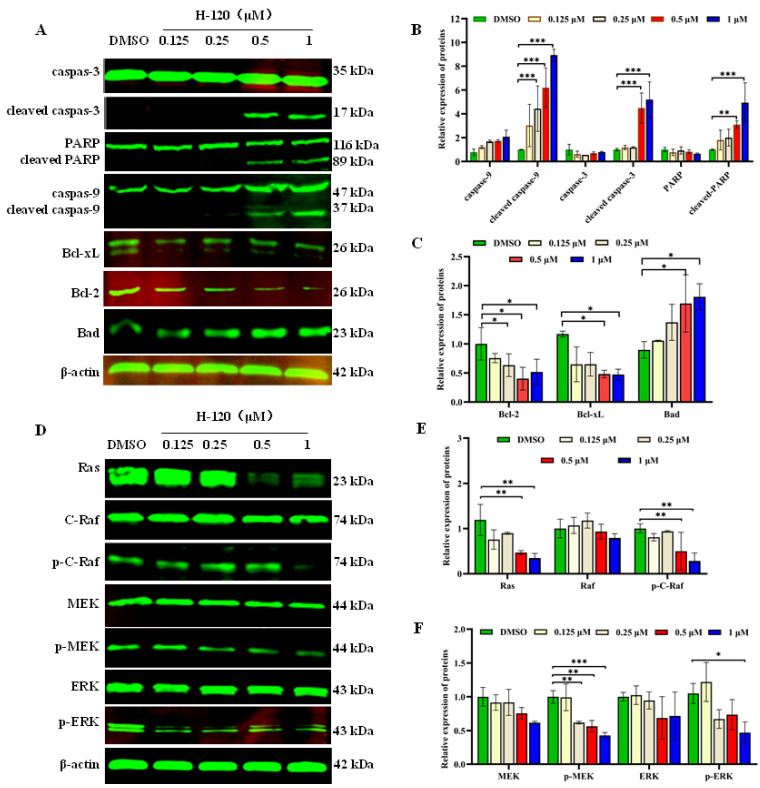
The effects of **H-120** on the mitochondrial apoptotic and MAPK signaling pathways. (**A**) The expressions of caspase-3, cleaved caspase-3, caspase-9, cleaved caspase-9, PARP, cleaved PARP, Bcl-xL, Bcl-2, and Bad were measured by Western blotting. (**B**,**C**): Quantification of relative protein expression levels of caspase-3, cleaved caspase-3, caspase-9, cleaved caspase-9, PARP, cleaved PARP, Bcl-xL, Bid, Bcl-2, and Bad, with β-actin used as loading control. (**D**) The expressions of Ras, MEK, p-MEK, ERK, and p-MRK were measured by Western blotting (**E**,**F**): Quantification of relative protein expression levels of Ras, MEK, p-MEK, ERK, and p-MRK, with β-actin used as loading control. Data are represented as mean ± SD (n = 3, * *p* < 0.05, ** *p* < 0.01, *** *p* <0.001, vs. the DMSO group).

To study the effect of **H-120** on apoptosis proteins, we treated HEL cells with various concentrations of **H-120**. The results showed that Bcl-2 and Bcl-xL were significantly down-regulated in HEL cells treated with **H-120**. But the Bad, cleaved caspase-9, cleaved caspase-3, and cleaved PARP were up-regulated considerably in HEL cells treated with **H-120** (Figure 4A–C). 

### 2.7. H-120 Regulates the ERK/Ras/Raf/MEK Signal Pathway in HEL Cells

To assess whether the effect of **H-120** on HEL cells is involved in the inactivation of Ras/Raf/MEK/ERK/MAPK signaling pathway, we tracked this pathway bottom-up in HEL cells treated with **H-120** using Western blotting. The results showed that **H-120** treatment could significantly reduce the expressions of Ras, phosphorylated Raf, MEK, and ERK in HEL cells (Figure 4D–F).

### 2.8. H-120 Inhibits STAT3/c-Myc Signal Pathway

**Figure 5:**
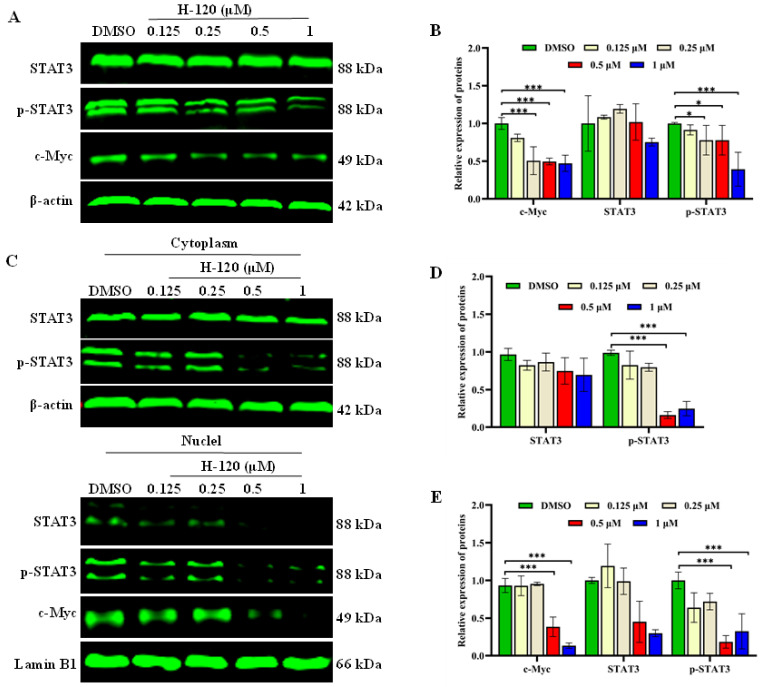
The effects of **H-120** on STAT3/c-Myc pathway. (**A**) The expression of STAT3, p-STAT3, and c-Myc were measured by Western blotting. (**B**) Quantification of relative protein expression levels of STAT3, p-STAT3, c-Myc, and β-actin was used as loading control; (**C**) Isolating cytoplasmic and nuclear proteins and the expression of STAT3, p-STAT3, and c-Myc were measured by Western blotting. (**D**,**E**): Quantification of relative protein expression levels of STAT3, p-STAT3, c-Myc, and β-actin was used as loading control for cytoplasmic protein; Lamin B1 was used as loading control for nuclear protein. Data are represented as mean ± SD (n = 3, * *p* < 0.05, *** *p* <0.001, vs. the DMSO group).

To examine the effect of **H-120** on the p-STAT3 and c-Myc, we checked the p-STAT3 and c-Myc levels in HEL cells treated with **H-120**. The results showed that they were significantly down-regulated in a dose-dependent manner. STAT3 acts through its phosphorylation and enters nuclear. The nuclear–cytoplasmic separation experiment was conducted. The results showed that the phosphorylated form of STAT3 did diminish when cells were treated with **H-120** in nuclear. Notably, the expression of c-Myc in the nucleus was also drastically reduced (Figure 5A–E). 

### 2.9. Anti-Erythroleukemia Activity of H-120 In Vivo

**Figure 6:**
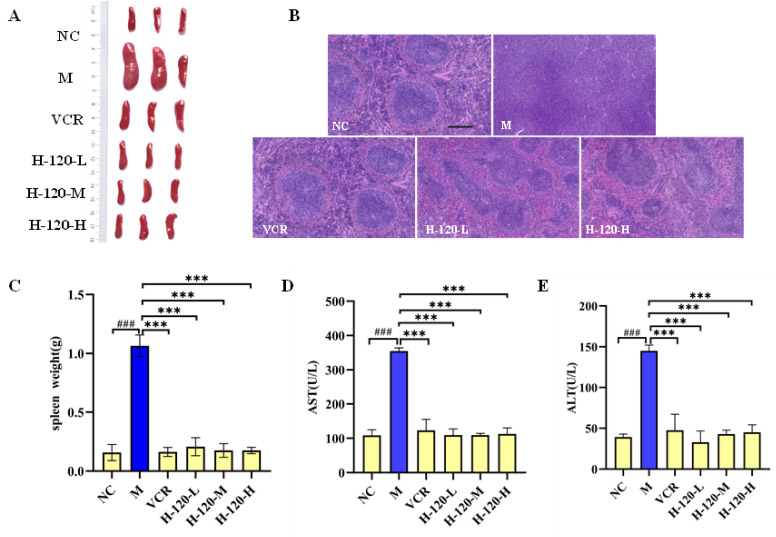
Therapeutic effect of **H-120** on erythroleukemia in vivo. (**A**) The spleens of mice; (**B**) H&E staining picture of mouse spleen (magnification: 100×; bar=200 μm). (**C**) Statistical analysis of spleen weight of mice after treatment with **H-120**. (**D**,**E**) Statistical chart of AST and ALT indicators. Data are represented as mean ± SD (n = 3; ^###^ *p* < 0.001, vs. M; *** *p* < 0.001, vs. M).

**Figure 7:**
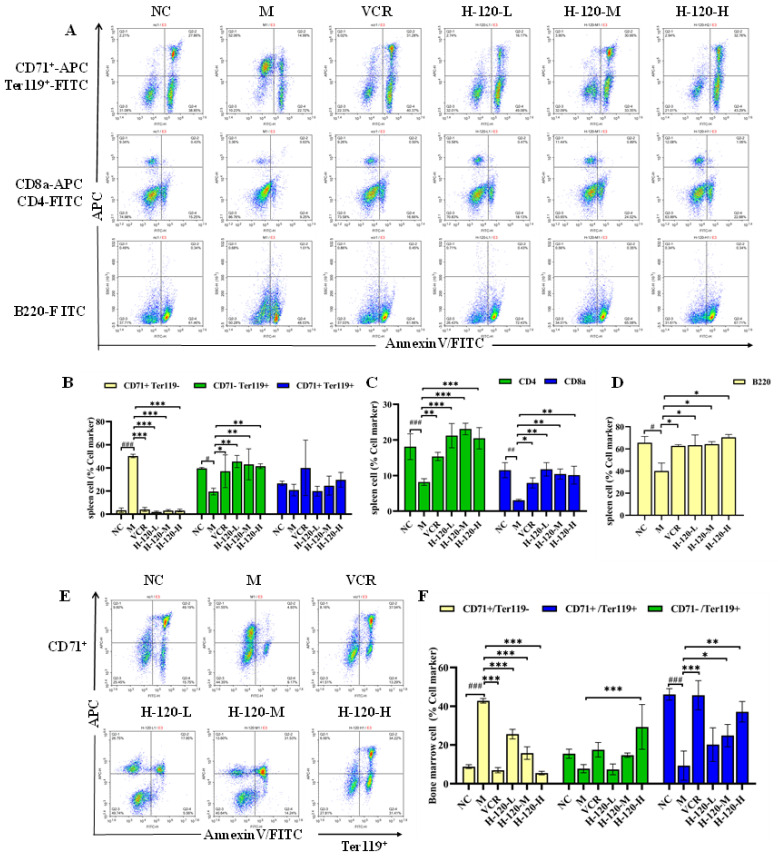
Effect of **H-120** on the expression of surface antigens CD71, Ter119, B220, CD4, and CD8a in spleen cells and bone marrow cells of erythroleukemia mice induced by F-MuLV. (**A**) Flow cytometry was used to analyze the effect of **H-120** on the expression of spleen cell surface antigens CD71, Ter119, B220, CD4, and CD8a. (**B**–**D**) Statistical map of surface antigens CD71, Ter119, B220, CD4, and CD8a and expression of spleen cells in mice. (**E**) Flow cytometry was used to analyze the effect of **H-120** on the expression of mouse bone marrow cell surface antigens CD71 and Ter119. (**F**) Statistical map of surface antigens CD71 and Ter119 and expression of bone marrow cells in mice. Data are represented as mean ± SD (n = 3; ^#^ *p* < 0.05, ^##^ *p* < 0.01, ^###^ *p* < 0.001, vs. M; * *p* < 0.05, ** *p* < 0.01, *** *p* < 0.001, vs. M).

We used the F-MuLV-induced leukemia mice to determine the anti-leukemia activity of **H-120**. The results indicated that the spleen weight of the **H-120** treatment mice significantly declined compared with the model mice. The effect of **H-120** treatment is similar to that in the vincristine (VCR) treatment group (Figure 6A,C). In addition, H&E staining showed a large number of basophilic leukemic cells in the spleen of the model group, and the normal structure was destroyed compared with the normal group. However, the number of leukemic cells in the spleens of the VCR and **H-120** groups decreased significantly, and the structures of the spleens were significantly restored (Figure 6B). Encouragingly, the levels of liver index, alanine aminotransferase (ALT), and aspartate aminotransferase (AST) were significantly lower in the **H-120** treatment group than in the model group (Figure 6D,E). The renal index (URA, CREA, and UA), cardiac index (CK-MB), triglycerides (TG), and total cholesterol (TC) of all groups were within the normal range, and the results suggest that **H-120** has no effects on the renal and cardiac functions of erythroleukemia mice (Supplementary Figure S1). The CD71 and Ter119 have been broadly used to reflect mice’s dynamic maturation of erythrocytes. We tested CD71 and Ter119 expression in spleen cells and bone marrow cells by using flow cytometry. The result suggested that the maturity of erythrocytes CD71^-^Ter119^+^ significantly increased in spleen cells and bone marrow cells when treated with **H-120** compared with the model group; furthermore, the effect of **H-120** was close to that of VCR treatment, indicating that **H-120** can promote erythroid differentiation into the maturation erythrocytes (Figure 7A,B,E,F). Moreover, the proportions of CD4, CD8a, and B220 cells in erythroleukemia spleen also increased significantly in the **H-120** treatment group, indicating that **H-120** effectively stimulates the immune cells in erythroleukemia mice (Figure 7A,C,D).

## 3. Discussion

Due to the rarity of the AEL (2–5% of all leukemias) with uncertain mutations in genes and abnormal changes at the molecular level, there are no standardized treatment methods in clinical practice [7]. Small molecular compounds are the main body of biological agents, and the mechanism of inducing apoptosis of cancer cells has been deeply studied. In this study, we found that H-120 inhibited leukemic HEL cells. 

Intact components of cell cycle arrest checkpoints are potential targets for novel antineoplastics [24,25]. When cell cycle checkpoints are not completed, cell cycle arrest will occur. The G2/M checkpoint is the final opportunity to repair damaged DNA prior to mitosis [26]. The upregulation of p53 expression can effectively impede tumor cell cycle progression, leading to subsequent G2/M arrest and apoptosis [26]. CDC25C serves as a crucial regulator in orchestrating the G2 to mitotic transition of the cell cycle and is involved in monitoring DNA damage repair processes [27]. Its downregulation through phosphorylation can induce cell arrest in the G2/M phase in response to DNA damage, mediated by p53 signaling. Meanwhile, the deletion of Cyclin B1 has been shown to inhibit tumor cell proliferation and decrease apoptosis. Cyclin B1 forms a complex with CDC2, the activation and maintenance of which are facilitated by CDC25C, thereby remaining active during the G2/M phase transition and progression. Our results indicate that H-120 significantly activates p53, reduces the phosphorylation of CDC25C, and diminishes the expression of Cyclin B1. Consequently, treated HEL cells were arrested in the G2/M phase.

As an essential participant in apoptosis, mitochondria is of great significance for cell division and differentiation as well as participation in apoptosis [28,29]. Its apoptotic mechanism is almost a dynamic equilibrium maintained by the Bcl-2 family [27]. The anti-apoptotic members of the Bcl-2 family, such as Bcl-2 (B cell leukemia or lymphoma gene number2) and Bcl-xL (B cell lymphoma-extra-large), are frequently overexpressed in neoplasia to inhibit apoptotic cell death during tumorigenesis [27]. Our study has proved that H-120 can cause a decrease in mitochondrial membrane potential in HEL cells. In addition, H-120 could down-regulate the expression of anti-apoptotic proteins Bcl-2 and Bcl-xL and upregulate the expression of pro-apoptotic protein Bad. Our results implied that H-120 induced mitochondrial damage of HEL cells, resulting in the release of cytochrome C from the mitochondria into cytosol. Caspases are a family of evolutionary conserved cysteine-dependent endoproteases that hydrolyze their substrates after specific aspartic acid residues [30]. The apoptotic caspases can be further sub-categorized into initiator and executioner caspases [31]. The caspases-9 have domain architecture akin to the inflammatory caspases. However, their function is to initiate apoptosis by activating the executioner caspases-3 [32,33]. PARP is the substrate of caspase-3 and is closely related to the integrity of DNA [33,34]. This study showed that H-120 upregulated the cleaved Caspase-9, cleaved caspase-3 and cleaved PARP. These results suggest that the apoptosis of HEL cells may be accomplished by the loss of mitochondrial membrane potential (MMP) caused by H-120, which activates the caspase cascade reaction and hydrolyzes PARP to make DNA fragmentation.

Unrestricted leukemia proliferation and decreased sensitivity to apoptotic-inducing agents and chemoresistance are typically associated with activating pro-survival pathways [11]. Ras is a small GTP-binding protein [35,36,37,38]; the activity of its protein is regulated by conformational state. When Ras is active GTP is bound. Active Ras can then bind Raf on the Ras-binding domain (BD) present in Raf. This results in the translocation of Raf to the cell membrane then activated by phosphorylation and dephosphorylation [39]. Activated Raf can interact with Ras, resulting in the activation of Raf-1 [40]. Raf-1 interacts with BCR-ABL to change the distribution of BAG on the mitochondrial membrane, thus preventing the apoptosis of hematopoietic cells with malignant proliferation. We found that H-120 significantly down-regulated the expression of Ras and p-C-Raf. In addition, Raf-1 can phosphorylate and inactivate Bad, which prevents cancer cells from normal apoptosis [41], H-120 can upregulate the expression of Bad. Therefore, we speculate that H-120 may not only restore the apoptosis-promoting ability of Bad from the mitochondrial pathway but also inhibit Raf-1 by inhibiting the activation of Ras, thus restoring the biological activity of Bad and causing HEL apoptosis. Raf is an effective activator of MEK, and ERK (extracellular signal-regulated kinase) is the main physiological substrate of MEK. Stimulation of Raf activates MEK1 and ERK, resulting in phosphorylation of transcription factors, proliferation, and inhibition of apoptosis [37,39]. Our findings showed that H-120 could significantly down-regulate the expression of Ras and the phosphorylated expression of MEK and ERK. Furthermore, Zehua et al. reported that disruption of the MEK/ERK/c-Myc central metabolic axis within tumor ECs is sufficient to halt tumor growth; inhibiting this central metabolic axis has positive implications for tumor ECs treatments [42]. c-Myc as a downstream effector of ERK signalling, and ERK may increase the transcriptional activity of c-Myc by directly phosphorylating Ser62 and regulating c-Myc horizontally after transcription [43,44]. Our investigation indicated that c-Myc expression was significantly decreased in H-120 tread HEL cells, which suggest that H-120 may disrupt the MEK/ERK/c-Myc biogenetic axis in HEL cell to halt its growth. In addition, the anti-erythroleukemia effect of H-120 may be related to the inhibition of the activation of STAT3/c-Myc signal pathway. Signal transducer and activator of transcription 3 (STAT3) is an important transcription factor [45]. When STAT3 was activated, phosphorylated STAT3 dimerized and translocated into the nucleus. Then it bonded to consensus response elements in the promoters of target genes, regulating the transcription of several genes associated with cellular proliferation (such as c-Myc, Bcl-2 and cyclins) and survival (such as Bcl-xL) [46]. We found that H-120 could significantly reduce the expression of p-STAT3 and c-Myc. Moreover, the p-STAT3 in the nucleus and cytoplasm of HEL cells treated with H-120 significantly decreased with the increase of H-120 concentration, and the content in the nucleus was substantially lower than that in the cytoplasm. These results indicated that H-120 suppressed STAT3 phosphorylation and activation in the cytoplasm. Obstructing STAT3 dimerization and inability to transfer the nucleus normally, and cannot be involved in activation of the c-Myc gene by directly binding to a site overlapping with the c-Myc E2F binding site in the c-Myc gene P2 promoter. Jihui Guo et al. [47] report that activation of STAT3 also stimulates cyclinB1 expression; it modulates G2/M phase checkpoint by regulating gene expressions of cyclinB1 and Cdc2 via E2F. Therefore, H-120 may also down-regulate the expression of cyclinB1 by inhibiting STAT3, thus arresting HEL cells in the G2 phase.

## 4. Materials and Methods

### 4.1. Reagents

Vincristine (VCR) was purchased from Wanle Pharmaceutical (Shenzhen, China). Roswell Park Memorial Institute 1640 (RPMI-1640, Gibco) was purchased from Thermo Fisher Scientific (Shanghai, China). Fetal bovine serum (FBS) was purchased from VACCA Biologics LLC. (Murphysboro, IL, United States). Dimethyl sulfoxide (D8371), bovine serum albumin (A8020), BCA protein assay kit (PC0020), thiazolyl blue tetrazolium bromide (M8180), and color mixed protein marker (PR 1920) was obtained from Solarbio Life Sciences (Beijing, China). An annexin V-FITC apoptosis detection kit was purchased from BD (Franklin Lakes, NJ, United States, Cat. No. 556547). A JC-1 mitochondrial membrane potential detection kit (C2006), Hoechst staining kit (C0003), reactive oxygen species assay kit (S0033), SDS-PAGE gel preparation kit (P0012AC), cell lysis buffer (P0013), SDS-PAGE sample loading buffer, 5× (P0015L), and transfer buffer (P0021B) were purchased from Beyotime Biotechnology (Shanghai, China). PARP (9542S), caspase-3 (9662S), caspase-9 (9502S), CDC25C (4688S), p-CDC25C (4901T), MEK1/2 (87276T), p-MEK1/2 (9154T), p53 (#2527), and Anti-rabbit IgG (#7074) were purchased from Cell Signaling Technology. β-actin (AF7018), C-Raf (AF6065), and p-C-Raf (AF3065) were purchased from Affinity Biosciences, United States. CyclinB1 (ab32053), STAT3 (ab119352), p-STAT3 (ab76315), c-Myc (ab32072), Ras (ab52939), ERK (ab184699), and p-ERK (ab32538) were purchased from Abcam (Cambridge, United Kingdom). CD71, Ter119, CD8a, CD4, and B220 were purchased from BD Biosciences. Lamin B1 (ET1606-27) was purchased from Huabio (Hangzhou, China).

### 4.2. Synthesis of Compounds

2-(2-Chloro-5-methyl-pyrimidin-4-ylamino)-thiophene-3-carboxylic acid methylamide (**A1**)

2,4-Dichloro-5-methylpyrimidine (1.296 g, 8 mmol), and 2-amino-*N*-methylthiophene-3-formamide (1.373 g, 8.8 mmol) were dissolved in anhydrous EtOH (40 mL). The solution was added with NaHCO_3_ (739 mg, 8.8 mmol) and anhydrous EtOH (40 mL). The mixture was refluxed for 12 h. The solution was cooled at room temperature, and the crude product was filtered. The filter was washed with EtOH to obtain yellow solids (1.218 g, 54% yield). ^1^H NMR (400 MHz, DMSO-*d_6_*) δ 12.73 (s, 1H), 8.48 (s, 1H), 8.22 (s, 1H), 7.47 (d, *J* = 6.0 Hz, 1H), 7.06 (d, *J* = 6.0 Hz, 1H), 2.82 (d, *J* = 4.4 Hz, 3H), 2.20 (s, 3H); ^13^C NMR (100 MHz, DMSO-*d_6_*) δ 166.5, 156.9, 156.8, 156.6, 146.5, 122.9, 117.4, 115.2, 115.1, 26.3, 12.8.

2-[5-Methyl-2-(4-nitro-phenylamino)-pyrimidin-4-ylamino]-thiophene-3-carboxylic acid methylamide (**B1**)

Compound **A1** (1.41 g, 5 mmol) was added to TFE (Trifluoroethanol, 20 mL). The solution was added with 4-nitroaniline (690 mg, 5 mmol) and TFA (Trifluoroacetic acid, 1.71 g, 15 mmol). The mixture was refluxed overnight under N_2_ atmosphere, cooled to room temperature, and extracted with EtOAc (100 mL × 3). The organic phase was washed with saturated NaHCO_3_ (50 mL × 3), dried over Na_2_SO_4_, and evaporated under vacuum to yield the crude product. The residue was purified by a silica-gel column using DCM (Dichloromethane):MeOH = 30:1 to obtain yellow solids (787 mg, 41% yield). ^1^H NMR (400 MHz, DMSO-*d_6_*) δ 12.41 (s, 1H), 9.91 (s, 1H), 8.39 (q, *J* = 4.4 Hz, 1H), 8.23–8.14 (m, 3H), 8.09–8.03 (m, 2H), 7.46 (d, *J* = 6.0 Hz, 1H), 7.03 (d, *J* = 6.0 Hz, 1H), 2.82 (d, *J* = 4.4 Hz, 3H), 2.19 (s, 3H); ^13^C NMR (100 MHz, DMSO-*d_6_*) δ 167.0, 157.7, 156.4, 156.2, 148.3, 147.7, 140.9, 125.8, 123.4, 118.7, 116.6, 114.6, 109.2, 26.7, 13.4.

2-[2-(4-Amino-phenylamino)-5-methyl-pyrimidin-4-ylamino]-thiophene-3-carboxylic acid methylamide (**C1**)

Compound **B1** (768 mg, 2 mmol) was resolved in MeOH (10 mL). The solution was added to Pd/C (86 mg, 10% wt) and stirred for 24 h at room temperature under H_2_ atmosphere. After filtering the mixture, the filtration was evaporated and recrystallized with methanol to yield yellow solids (507 mg, 72% yield). ^1^H NMR (400 MHz, DMSO-*d_6_*) δ 12.15 (s, 1H), 8.52 (s, 1H), 8.31 (s, 1H), 7.93 (s, 1H), 7.39 (d, *J* = 6.0 Hz, 1H), 7.27 (d, *J* = 8.4 Hz, 2H), 6.86 (d, *J* = 6.0 Hz, 1H), 6.53 (d, *J* = 8.4 Hz, 2H), 4.75 (s, 2H), 2.81 (d, *J* = 4.4 Hz, 3H), 2.10 (s, 3H); ^13^C NMR (100 MHz, DMSO-*d_6_*) δ 166.7, 159.3, 156.4, 155.7, 147.9, 144.4, 130.0, 123.2, 122.8, 115.6, 114.3, 113.4, 104.7, 26.2 12.8.

2-[5-Methyl-2-(4-propynoylamino-phenylamino)-pyrimidin-4-ylamino]-thiophene-3-carboxylic acid methylamide (**H-120**)

Compound **C1** (177 mg, 0.5 mmol) and DIEA (*N*,*N*-Diisopropylethylamine, 129 mg, 1 mmol) were resolved in DMF (*N*,*N*-Dimethylformamide, 4 mL). The solution was added with propiolic acid (35 mg, 0.5 mmol) and HATU (2-(7-Azabenzotriazol-1-yl)-*N*,*N*,*N*′,*N*′-tetramethyluronium hexafluorophosphate, 380 mg, 1 mmol). The mixture was stirred for 24 h at room temperature and extracted with EtOAc (50 mL × 3). The organic phase was washed with saturated NaCl (20 mL × 3), dried over Na_2_SO_4_, and evaporated under vacuum to yield the crude product. The residue was purified by a silica-gel column using DCM/MeOH = 30/1 to obtain light yellow solids (97 mg, 48% yield). ^1^H NMR (600 MHz, DMSO-*d*_6_) *δ*12.38 (s, 1H), 10.67 (s, 1H), 9.03 (s, 1H), 8.33 (m, 1H), 8.00 (s, 1H), 7.65 (d, *J* = 8.0 Hz, 2H), 7.47 (d, *J* = 8.0 Hz, 1H), 7.40 (d, *J* = 4.0 Hz, 1H), 6.91 (d, *J* = 4.0 Hz, 1H), 4.34 (s,1H), 2.78 (s, 3H), 2.10 (s, 3H). ^13^C NMR (150 MHz, DMSO-*d*_6_) *δ*166.2, 157.9, 155.7, 155.4, 149.3, 147.2, 137.31, 131.6, 122.5, 120.1, 119.9, 115.41, 113.3, 105.9, 78.7, 76.8, 25.8, 12.5.

2-((2-((4-Acrylamidophenyl)amino)-5-methylpyrimidin-4-yl)amino)-*N*-methylthiophene-3-carboxamide (**H-121**)

Compound **C1** (177 mg, 0.5 mmol) and DIEA (129 mg, 1 mmol) were resolved in DCM (5 mL). The solution was slowly added to the solution of DCM (1 mL) containing acryloyl chloride (45 mg, 0.5 mmol). The mixture was stirred for 24 h at room temperature and extracted with EtOAc (50 mL × 3). The organic phase was washed with saturated NaCl (20 mL × 3), dried over Na_2_SO_4_, and evaporated under vacuum to yield the crude product. The residue was purified by a silica-gel column using DCM/MeOH = 30/1 to obtain yellow solids (75 mg, 37% yield). ^1^H NMR (400 MHz, DMSO-*d_6_*) δ 10.30 (s, 1H), 10.03 (d, *J* = 5.2 Hz, 1H), 7.75 (d, *J* = 8.4 Hz, 2H), 7.64 (s, 1H), 7.27 (d, *J* = 8.4 Hz, 2H), 7.09 (d, *J* = 5.6 Hz, 1H), 6.50 (dd, *J* = 7.6, 9.6 Hz, 2H), 6.34–6.31 (m, 1H), 6.28 (dd, *J* = 5.6, 1.6 Hz, 1H), 6.08 (dd, *J* = 9.6, 9.2 Hz, 1H), 5.80 (dd, *J* = 9.6, 2.4 Hz, 1H), 2.80 (d, *J* = 4.8 Hz, 3H), 2.01 (s, 3H).

2-((2-Chloro-5-(trifluoromethyl)pyrimidin-4-yl)amino)-*N*-methylbenzamide (**A2**)

2,4-Dichloro-5-trifluoromethylpyrimidine (1.728 g, 8 mmol) was resolved in EtOH (20 mL). The solution was added with 2-amino-*N*-methylbenzamide (1.32 g, 8.8 mmol) and NaHCO_3_ (739 mg, 8.8 mmol) at room temperature. The mixture was heated to reflux and stirred overnight. The precipitate was filtered out and washed with EtOH to yield the compound as a yellow solid (1.452 g, 55% yield). ^1^H NMR (400 MHz, DMSO-*d_6_*) δ 12.06 (s, 1H), 8.86 (q, *J* = 4.4 Hz, 1H), 8.68 (s, 1H), 8.38 (d, *J* = 8.4 Hz, 1H), 7.78 (dd, *J* = 8.0, 1.2 Hz, 1H), 7.59 (td, *J* = 8.0, 1.2 Hz, 1H), 7.26 (td, *J* = 8.0, 1.2 Hz, 1H), 2.34 (d, *J* = 4.4 Hz, 3H); ^13^C NMR (400 MHz, DMSO-*d_6_*) δ 169.1, 162.7, 157.3, 156.9, 138.0, 132.1, 128.5, 124.9, 124.4, 122.8, 122.3, 107.9, 26.8.

*N*-Methyl-2-((2-((4-nitrophenyl)amino)-5-(trifluoromethyl)pyrimidin-4-yl)amino)benzamide (**B2**)

Compound **A2** (1.65 g, 5 mmol) was resolved in TFE (2,2,2-trifluoroethanol, 20 mL). The solution was added with 4-nitroaniline (828 mg, 6 mmol) and TFA (trifluoroacetic acid, 1.71 g, 15 mmol). The mixture was heated to reflux under a nitrogen atmosphere, then stirred overnight. The mixture was added with EtOAc (100 mL) and washed with saturated NaHCO_3_ (50 mL). The organic layer was dried over Na_2_SO_4_, filtered, and concentrated in vacuo to afford the crude compound. The residue was purified by a silica-gel column using DCM/MeOH = 30/1 to yield yellow solids (1.058 g, 49% yield). M.p > 250 °C; ^1^H NMR (400 MHz, DMSO-*d_6_*) δ 12.06 (s, 1H), 8.86 (q, *J* = 4.4 Hz, 1H), 8.68 (s, 1H), 8.38 (d, *J* = 8.4 Hz, 1H), 7.78 (dd, *J* = 8.0, 1.2 Hz, 1H), 7.59 (m, 1H), 7.26 (m, 1H), 2.34 (d, *J* = 4.4 Hz, 3H); ^13^C NMR (400 MHz, DMSO-*d_6_*) δ 169.1, 160.6, 156.8, 156.4, 146.7, 141.5, 138.7, 131.7, 128.5, 126.0, 125.1, 123.9, 123.8, 123.4, 119.3, 101.2, 26.7.; ESI-HRMS C_19_H_15_F_3_N_6_O_3_ ([M + Na]^+^): calcd 455.1056, found 455.1037.

2-((2-((4-Aminophenyl)amino)-5-(trifluoromethyl)pyrimidin-4-yl)amino)-*N*-methylbenzamide (**C2**)

Compound **B2** (864 mg, 2 mmol) was resolved in MeOH (10 mL). The solution was added with Pd/C (86 mg, 10% wt) and stirred for 24 h at room temperature under an H_2_ atmosphere. After filtering the mixture, the filtration was evaporated and recrystallized with methanol to yield yellow solids (611 mg, 76% yield). ^1^H NMR (400 MHz, DMSO-*d_6_*) δ 11.30 (s, 1H), 9.42 (s, 1H), 8.72 (d, *J* = 4.4 Hz, 1H), 8.36 (m, 2H), 7.70 (d, *J* = 7.6 Hz, 1H), 7.47–7.07 (m, 4H), 6.51 (d, *J* = 8.8 Hz, 2H), 4.89 (s, 2H), 2.78 (d, *J* = 4.4 Hz, 3H); ^13^C NMR (100 MHz, DMSO-*d_6_*) δ 169.3, 161.9, 160.2, 156.3, 145.5, 139.3, 132.0, 128.23, 126.6, 125.7, 123.7, 122.6, 118.8 (q, *J* = 5.2 Hz), 116.1, 114.7, 114.2, 26.7; ESI-HRMS C_19_H_17_F_3_N_6_O ([M + H]^+^): calcd 403.1488, found 403.1478.

2-((2-((4-((2-Methoxy-3,4-dioxocyclobut-1-en-1-yl)amino)phenyl)amino)-5-(trifluoromethyl)pyrimidin-4-yl)amino)-*N*-methylbenzamide (**D1**)

Compound **C2** (804 mg, 2 mmol) was resolved in DMF (10 mL). The solution was added with dimethyl squarate (284 mg, 2 mmol) and DIEA (258 mg, 2 mmol). The mixture was stirred at room temperature for 12 h. The mixture was extracted with EtOAc (100 mL × 3), and the combined organic phase was washed with saturated brine (50 mL × 3). The organic layer was dried over magnesium sulfate, filtered, and concentrated in vacuo to afford the crude compound. The residue was purified by a silica-gel column using DCM/MeOH = 30/1 to yield white solids (635 mg, 62% yield). M.p: 234.8–235.6 °C; IR (KBr pellet): v 3229.42, 1804.30, 1703.14, 1619.83, 1539.50, 1515.70, 1018.84, 828.43, 760.00; ^1^H NMR (400 MHz, DMSO-*d_6_*) δ 11.32 (s, 1H), 10.72 (s, 1H), 9.87 (s, 1H), 8.75 (s, 1H), 8.44 (s, 1H), 7.68 (m, 3H), 7.50 (t, *J* = 7.6 Hz, 1H), 7.31–7.16 (m, 4H), 4.39 (s, 3H), 2.79 (d, *J* = 4.4 Hz, 3H); 13C NMR (125 MHz, DMSO-*d_6_*) δ 186.6, 170.2, 166.8, 165.8, 155.4, 150.3, 148.9, 145.4, 145.2, 140.5, 131.2, 129.0, 125.3, 122.7, 121.0, 119.2 (q, *J* = 5.2 Hz), 118.5, 117.2, 116.4, 68.8, 41.4. ESI-HRMS C_24_H_19_F_3_N_6_O_4_ ([M + H]^+^): calcd 513.1492, found 513.1492.

2-((2-((4-((2-(((1*R*,3*R*,5*S*)-Adamantan-1-yl)amino)-3,4-dioxocyclobut-1-en-1-yl)amino)phenyl)amino)-5-(trifluoromethyl)pyrimidin-4-yl)amino)-*N*-methylbenzamide (**H-122**)

Compound **D1** (528 mg, 1 mmol) was resolved in DMF (10 mL). The solution was added with (1*R*,3*R*,5*S*)-adamantan-1-amine (181 mg, 1.2 mmol) and DIEA (129 mg, 1 mmol). The mixture was stirred at room temperature for 8 h. The mixture was extracted with EtOAc (50 mL × 3) and the combined organic phase was washed with saturated brine (20 mL × 3). The organic layer was dried over Na_2_SO_4_, filtered, and concentrated in vacuo to afford the crude compound. The residue was purified by a silica-gel column using DCM/MeOH = 30/1 to yield yellow solids (246 mg, 39% yield). ^1^H NMR (400 MHz, DMSO-*d_6_*) δ 11.32 (s, 1H), 9.85 (s, 1H), 9.69 (s, 1H), 8.75 (d, *J* = 4.4 Hz, 1H), 8.43 (s, 1H), 7.86 (d, *J* = 8.0 Hz, 1H), 7.72 (d, *J* = 8.4 Hz, 1H), 7.67–7.49 (m, 4H), 7.39 (d, *J* = 8.8 Hz, 2H), 7.18 (t, *J* = 7.6 Hz, 1H), 4.22 (d, *J* = 7.6 Hz, 1H), 2.79 (d, *J* = 4.4 Hz, 3H), 2.04–1.58 (m, 14H).

2-(2-Chloro-5-trifluoromethyl-pyrimidin-4-ylamino)-thiophene-3-carboxylic acid methylamide (**A3**)

2,4-Dichloro-5-trifluoromethylpyrimidine (2.16 g, 10 mmol) was added to a stirred solution of 2-amino-*N*-methylthiophene-3-carboxamide (1.716 g, 11 mmol) and NaHCO_3_ (924 mg, 11 mmol) in anhydrous EtOH (30 mL) at room temperature. The resulting mixture was heated to reflux and stirred overnight before cooling at room temperature. The precipitate was filtered out and washed with water to yield the compound as a yellow solid (1.344 g; 40% yield). M.p: 136.3–138.2 °C; ^1^H NMR (400 MHz, DMSO-*d_6_*) δ 13.68 (s, 1H), 8.75 (s, 1H), 8.59 (q, *J* = 4.8 Hz, 1H), 7.51 (d, *J* = 6.0 Hz, 1H), 7.19 (d, *J* = 6.0 Hz, 1H), 2.82 (d, *J* = 4.4 Hz, 3H); ^13^C NMR (400 MHz, DMSO-*d_6_*) δ 166.1, 162.1, 157.2, 157.1, 153.3, 144.9, 122.9, 119.3, 116.7, 107.3, 26.3.; ESI-HRMS C_11_H_8_ClF_3_N_4_OS ([M + Na]^+^): calcd 358.9951, found 358.9949.

2-[2-(4-Nitro-phenylamino)-5-trifluoromethyl-pyrimidin-4-ylamino]-thiophene-3-carboxylic acid methylamide (**B3**)

To a solution of compound **A3** (2.016 g, 6 mmol) in TFE (2,2,2-trifluoroethanol, 24 mL), 4-nitroaniline (911 mg, 6.6 mmol) and TFA (trifluoroacetic acid, 2.052 g, 18 mmol) were added. The resulting mixture was heated to reflux under a nitrogen atmosphere and stirred overnight before cooling to room temperature. The mixture was added with EtOAc (100 mL) and washed with saturated NaHCO_3_ (3 × 50 mL). The organic layer was dried over magnesium sulfate, filtered, and concentrated in vacuo to afford the crude compound. The residue was purified by a silica-gel column using DCM/MeOH = 30/1 to yield the product as a yellow solid (1.13 g; 43% yield). M.p > 250 °C; ^1^H NMR (400 MHz, DMSO-*d_6_*) δ 13.13 (s, 1H), 10.40 (s, 1H), 8.62 (s, 1H), 8.47 (d, *J* = 4.4 Hz, 1H), 8.24 (d, *J* = 9.2 Hz, 2H), 8.05 (d, *J* = 9.2 Hz, 2H), 7.48 (d, *J* = 6.0 Hz, 1H), 7.13 (d, *J* = 6.0 Hz, 1H), 2.81 (d, *J* = 4.4 Hz, 3H); ^13^C NMR (100 MHz, DMSO-*d_6_*) δ 166.2, 160.4, 156.1, 153.2, 146.3, 145.6, 142.0, 125.1, 123.0, 120.3, 117.7, 115.7, 101.2, 100.9, 26.2; ESI-HRMS C_17_H_13_F_3_N_6_O_3_S ([M + Na]^+^): calcd 461.0620, found 461.0606.

2-[2-(4-Amino-phenylamino)-5-trifluoromethyl-pyrimidin-4-ylamino]-thiophene-3-carboxylic acid methylamide (**C3**)

To a solution of compound **B3** (876 mg, 2 mmol) in methanol (20 mL), Pd/C (263 mg, 10% wt) was added. The mixture was stirred at room temperature under a hydrogen atmosphere for 24 h. The solution was filtered with celite, and the filtration was evaporated under vacuum. The crude solid was recrystallized with methanol to yield compound **3** as a yellow solid (0.286 g; 35% yield). M.p > 250 °C; ^1^H NMR (400 MHz, DMSO-*d_6_*) δ 12.82 (s, 1H), 9.38 (s, 1H), 8.37 (s, 2H), 7.58–6.88 (m, 4H), 6.57 (d, *J* = 7.6 Hz, 2H), 4.97 (s, 2H), 2.80 (s, 3H); ^13^C NMR (100 MHz, DMSO-*d_6_*) δ 166.2, 156.3, 153.2, 146.2, 145.9, 138.4, 134.4, 130.0, 126.5, 123.8, 122.8, 117.2, 114.9, 114.8, 26.2; ESI-HRMS C_17_H_15_F_3_N_6_OS ([M + H]^+^): calcd 409.1052, found 409.1045.

2-{2-[4-(2-Methoxy-3,4-dioxo-cyclobut-1-enylamino)-phenylamino]-5-trifluoromethyl-pyrimidin-4-ylamino}-thiophene-3-carboxylic acid methylamide (**D2**)

To a solution of compound **C3** (1.224 g, 3 mmol) in DMF (15 mL), dimethyl squarate (426 mg, 3 mmol) and DIEA (516 mg, 4 mmol) were added. The mixture was stirred at room temperature overnight. The mixture was extracted with EtOAc (150 mL × 2), and the combined organic phase was washed with saturated brine (100 mL × 3). The organic layer was dried over Na_2_SO_4_, filtered, and concentrated in vacuo to afford the crude compound. The residue was purified by a silica-gel column using DCM/MeOH = 30/1 to yield white solids (886 mg, 57% yield). M.p: 233.1–235.0 °C; ^1^H NMR (400 MHz, DMSO-*d_6_*) δ 12.94 (s, 1H), 10.77 (s, 1H), 9.79 (d, *J* = 0.8 Hz, 1H), 8.55–8.35 (m, 2H), 7.62 (s, 2H), 7.38 (m, 3H), 7.01 (s, 1H), 4.39 (s, 3H), 2.80 (d, *J* = 3.6 Hz, 3H); ^13^C NMR (125 MHz, DMSO-*d_6_*) δ 187.5, 170.5, 167.2, 155.6, 153.0, 150.3, 145.1, 142.6, 136.8, 132.0, 128.6, 120.8, 119.1, 118.3, 116.4, 114.0, 112.2, 68.8, 41.0; ESI-HRMS C_22_H_17_F_3_N_6_O_4_S ([M + H]^+^): calcd 519.1056, found 519.1061.

2-((2-((4-((2-(((1*R*,3*R*,5*S*)-Adamantan-1-yl)amino)-3,4-dioxocyclobut-1-en-1-yl)amino)phenyl)amino)-5-(trifluoromethyl)pyrimidin-4-yl)amino)-*N*-methylthiophene-3-carboxamide (**H-123**)

Compound **D2** (518 mg, 1 mmol) was resolved in DMF (10 mL). The solution was added with (1*R*,3*R*,5*S*)-adamantan-1-amine (181 mg, 1.2 mmol) and DIEA (129 mg, 1 mmol). The mixture was stirred at room temperature for 8 h. The mixture was extracted with EtOAc (50 mL × 3), and the combined organic phase was washed with saturated brine (20 mL × 3). The organic layer was dried over Na_2_SO_4_, filtered, and concentrated in vacuo to afford the crude compound. The residue was purified by a silica-gel column using DCM/MeOH = 30/1 to yield yellow solids (236 mg, 37% yield). ^1^H NMR (400 MHz, DMSO-*d_6_*) δ 12.93 (s, 1H), 9.95 (s, 1H), 9.77 (s, 1H), 8.44 (d, *J* = 24.8 Hz, 2H), 7.96 (s, 1H), 7.72–7.42 (m, 5H), 7.02 (s, 1H), 4.23 (d, *J* = 4.8 Hz, 1H), 2.80 (d, *J* = 3.6 Hz, 3H), 2.04–1.60 (m, 14H)

### 4.3. Cell Culture

HEL (erythroleukemia), K562 (Chronic Myeloid Leukemia), Jurkat (acute T lymphoblastic leukemia), and HL7702 (normal hepatic cell line) were purchased from ATCC, Manassas, VA, United States. The cells were cultured in RPMI 1640 medium supplemented with 5% FBS at 37 °C in a CO_2_ incubator (5% CO_2_ and 95% air, 95% humidity), as per standard conditions of passage.

### 4.4. Cell Viability Assays

The logarithmically grown HEL cells were seeded in a 96-well culture plate at a density of 8000 cells per well and treated with different concentrations of **H-120** (0.125 μM, 0.25 μM, 0.5 μM, and 1 μM) or 0.1% DMSO (control). After 24 h, 48 h, and 72 h treatment, the cells were added to 10 μL of MTT reagent for 4 h, and then added to 100 μL of triple cleavage fluid (SDS 10 g, isobutanol 5 mL and 10M HCl 0.1 mL, dissolved with ddH_2_O into 100 mL solution). After using a microscope to observe the completely dissolved crystal, the cells’ optical absorbance value was measured at 570 nm using an enzyme-labeling instrument (BioTek, Winooski, VT, United States). Inhibition rate = (OD value of DMSO group-OD value of experimental group)/OD value of DMSO group × 100%.

### 4.5. Cell Growth Curve Determination

The logarithmically grown HEL cells were collected and planted in 96-well plates at a density of 8000 cells per well. After the cell state was stabilized, HEL cells were treated with **H-120** solution (0.125 μM, 0.25 μM, 0.5 μM, 1 μM), and then the OD value was measured at 570 nm in different periods of time. The growth curves were plotted.

### 4.6. Apoptosis and Cell Cycle Analyses

For the apoptosis detection assay, the logarithmically grown HEL cells were treated with **H-120** solution (0.125 μM, 0.25 μM, 0.5 μM, and 1 μM) for 24 h, 48 h, and 72 h. Subsequently, the cells were collected and rinsed three times with pre-cooled PBS. Based on the instruction of the Annexin V-FITC apoptosis detection kit, the cells were resuspended in 50 μL of 1 × binding buffer and stained for 15 minutes. Then, the apoptosis rate was determined using flow cytometry (ACEA NovoCyte, San Diego, CA, United States). For cell cycle analysis, HEL cells were treated with the different concentrations of **H-120** (0.125 μM, 0.25 μM, 0.5 μM, 1 μM) for 24 h. Cells were collected, fixed with pre-cooled 70% ethanol, and kept at 4 °C for 4 h before being transferred to −20 °C overnight. Cells were stained with 0.5 mL of PI (propidium iodide, 50 µg/mL), RNase (5 µg/mL) inhibitor, and Triton × 100 (0.5 µg/mL) for 10 min at room temperature in the dark and then analyzed by flow cytometry.

### 4.7. Hoechst 33258 Staining

After subjecting HEL cells to **H-120** solution (0.125 μM, 0.25 μM, 0.5 μM, 1μM) for 24 h, the cells were collected, resuspended, and washed with pre-cooled PBS. Subsequently, the cells were fixed, stained, and washed according to the instructions provided by the Apoptosis-Hoechst 33258 Staining Kit. To prevent fluorescence interference, an appropriate volume of anti-fluorescence quenching sealing solution was added to the cells. The cells were then placed on a spare slide, covered with a coverslip, and immediately observed and photographed using a Nikonfluorescence microscope.

### 4.8. Mitochondrial Membrane Potential Assay

Cells (2 × 10^5^) were seeded in a 2 mL medium with different concentrations of **H-120** (0.125 μM, 0.25 μM, 0.5 μM, 1 μM). After being treated with **H-120** for 48 h, the cells were preincubated with JC-1 working solution for 20 min at 37 °C, 5% CO_2_. After incubation, the dye was removed, and the cells were washed two times with JC-1 [48]. Subsequently, the change in the membrane potential of treated cells concerning control cells was detected using a fluorescence microscope, and the proportion of cells exhibiting red or green fluorescence was documented.

### 4.9. Extraction of Nuclear Protein and Cytoplasmic Protein

HEL cells were collected after 24 h of **H-120** treatment and washed with PBS, and the supernatant was discarded. According to the instructions of the Nuclear and Cytoplasmic Protein Extraction Kit (Beyotime Biotechnology, Shanghai, China), we added cytoplasmic protein extraction reagent A equipped with PMSF (phenylmethanesulfonyl fluoride) and used vigorous vortexing at 2500 rpm for 5 s to suspend and disperse the cell precipitation entirely. After 15 min of an ice bath, we added cytoplasmic protein extraction reagent B. The sample was vigorously vortexed at 2500 rpm for 5 s, then centrifuged at 4 °C for 5 min at 12,000× *g*, and the supernatant was extracted to leave a precipitate. The supernatant was the cytoplasmic protein. For the precipitate, the cytosolic protein extraction reagent equipped with PMSF was added immediately, mixed, and then placed on ice. The highest-speed vigorous vortex was performed for 20 s every 2 min for 30 min, followed by centrifugation at 12,000× *g* at 4 °C for 10 min. Finally, the supernatant was taken as the nuclear protein.

### 4.10. Western Blotting Analysis

HEL cells after drug action were collected, added to and mixed with cell lysis buffer, and immunoprecipitated on ice for one hour before protein concentration was detected using a BCA (bicinchoninic acid) kit. Proteins (50 μg) were separated by sodium dodecyl sulfate-polyacrylamide gel electrophoresis and transferred to a PVDF (polyvinylidene fluoride) membrane (0.22 µM, Bio-Rad, Hercules, CA, USA) via wet transfer. Then, 5% bovine serum albumin closed the membrane for 1 h, and the membrane was washed three times with TBS (20 mM Tris-HCl) for 5 min each time. Membranes were allowed to incubate with the primary antibody overnight at 4 °C. The anti-rabbit antibody secondary antibody was incubated at room temperature for 2 h. Then, the membrane was washed with 1× TBST (20 mM Tris-HCl, 0.1% Tween 20) for 15 min. After washing, the fetal membrane was scanned with an infrared imaging scanner. β-actin was used as a loading control.

### 4.11. Animal Experiment

The NIH3T3 cells expressing the F-MuLV clone 57 vector were donated by Professor Ben-David Yaacov, Key Laboratory of Natural Product Chemistry, Chinese Academy of Sciences, Guizhou Province. In order to induce leukemia, BALB/c mice (male and females; Tongxin, Chongqing, China), aged one day, were administered a solitary intraperitoneal injection of F-MuLV. Then, infected neonates were weaned at 4 weeks and randomly divided into 6 groups: normal group (NC), model group (M), positive control group (VCR: 0.25 mg/kg), **H-120** low dose group (H-120-L: 1 mg/kg), **H-120** medium dose group (H-120-M: 2 mg/kg), and **H-120** high dose group (H-120-H: 4 mg/kg). After 5 weeks of virus infection, mice were treated with **H-120** intraperitoneally every other day for 3 weeks. The normal and model groups were given saline, and the control group was given vincristine intraperitoneally. Under the supervision of experienced staff, the leukemia mice were sacrificed humanely by cervical dislocation. Serum biochemical indexes of the mice were detected. The spleens of the mice were removed. A portion of the spleen was fixed in formaldehyde for 24 h, and paraffin was embedded and sliced. The effects of **H-120** on the normal physiological structure of the spleen were observed by H&E staining. Then, part of the spleen tissue was ground and prepared into a single-cell suspension, which was stained with CD71, Ter119, CD4, CD8a, and B220 antibodies for 30 min, respectively. The expression of markers on the spleen cell surfaces of mice was analyzed by flow cytometry.

### 4.12. Ethics Statement

All animal experiments were approved by the ethics committee of State Key Laboratory for Functions and Applications of Medicinal Plants, Guizhou Medical University with the approval number of 2200956.

### 4.13. Statistical Analysis

All data are presented as the mean ± standard deviation (SD) from at least three independent experiments. Statistical analysis was performed using Student’s *t*-test or one-way ANOVA in GraphPad Prism 8.0 (GraphPad Software, La Jolla, CA, USA) and SPSS 26.0 (IBM Corporation, Armonk, NY, USA). *p* < 0.05 was considered statistically significant.

## 5. Conclusions

**Figure 8:**
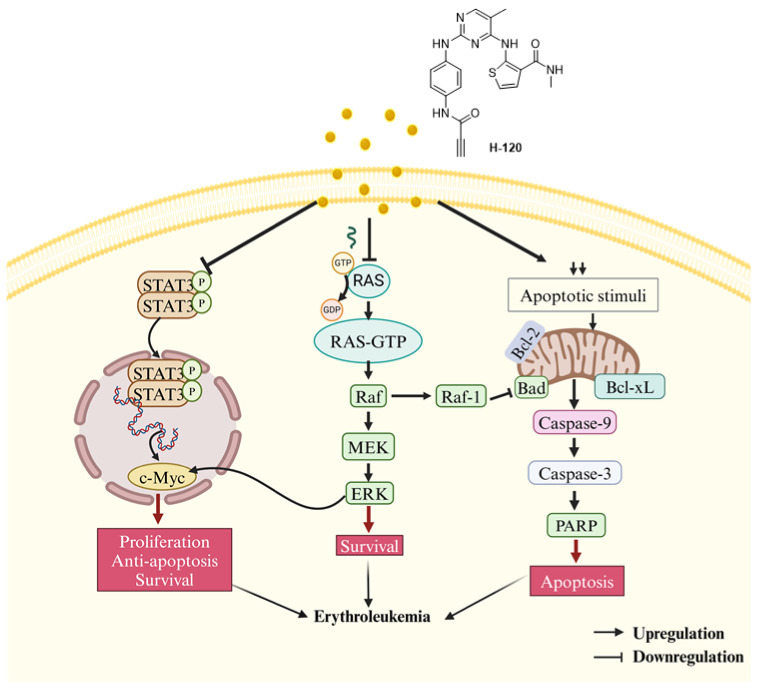
Schematic mechanisms of **H-120** apoptosis by regulating Ras/MER/ERK MAPK and STAT3/c-Myc signaling pathways.

**H-120** exerted excellent anti-leukemia activity both in vitro and in vivo. The compound predominantly displayed the highest cytotoxic potential in HEL cells. It induced apoptosis in HEL cells through the mitochondrial caspase-dependent pathway and arrested HEL cells in G2/M. Furthermore, **H-120** inhibited HEL cell proliferation by dual blockade of the Ras/Raf/MEK/ERK and STAT3/c-Myc pathways (Figure 8). Moreover, the compound effectively inhibited the progression of and protected liver function from leukemia in mice. Therefore, **H-120** may be a potential chemotherapeutic drug for leukemia patients.

## Data Availability

The data presented in this study are available upon request from the corresponding author.

## Supplementary Materials

The following supporting information can be downloaded at: https://www.mdpi.com/article/10.3390/molecules29071597/s1, Figure S1: Statistical chart of UREA, CREA, UA, TG, TC, and CK-MB indicators; Synthesis, nuclear magnetic, and high-resolution mass spectra of compounds: Figures S2–S8: ^1^H NMR spectrum of compounds **A1**, **B1**, **C1**, **H-120**, **H-121**, **H-122** and **H-123**; Figures S9–S12: ^13^C NMR spectrum of compounds **A1**, **B1**, **C1**, and **H-120**. Figure S13: ^13^C NMR spectrum of compound **H-120**. Figure S14: HSQC spectrum of compound **H-120**. Figure S15: HMBC spectrum of compound **H-120**. Figure S16: ^1^H-^1^H COSY spectrum of compound **H-120**. Figure S17: ^1^H NMR spectrum of compound **H-121**. Figure S18: ^1^H NMR spectrum of compound **H-122**. Figure S19: ^1^H NMR spectrum of compound **H-123**.
