# Supplementary material for: Design, Synthesis, and Anti-Leukemic Evaluation of a Series of Dianilinopyrimidines by Regulating the Ras/Raf/MEK/ERK and STAT3/c-Myc Pathways
Source: Molecules. 2024 Apr 3;29(7):1597. doi: 10.3390/molecules29071597 (PMC11013136; doi:10.3390/molecules29071597)
Supplement: Supplementary file 1 [file molecules-29-01597-s001.zip › molecules-2783934-supplementary.pdf]

Supplementary Material

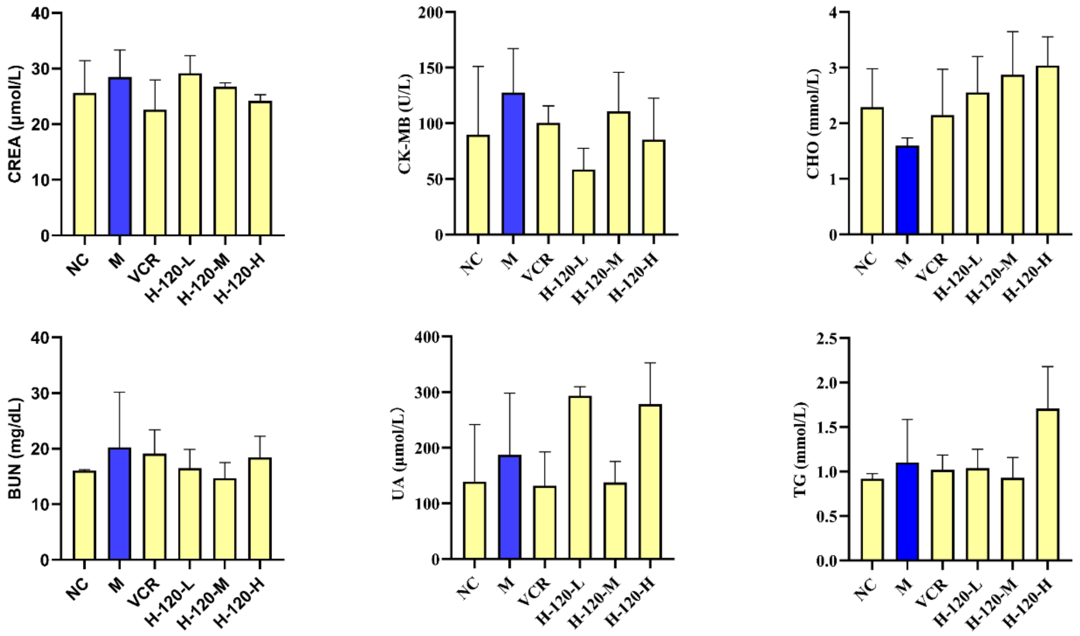

Supplementary Fig S1: Statistical chart of UREA, CREA, UA, TG, TC, and CK-MB indicators. (All indicators are within the normal range)

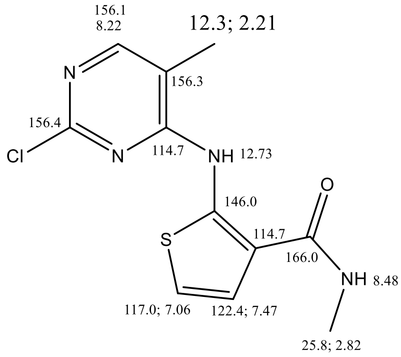

Supplementary Fig S2. compound **A1**：2-((2-chloro-5-methylpyrimidin-4-yl)amino)-N-methylthiophene-3-carboxamide

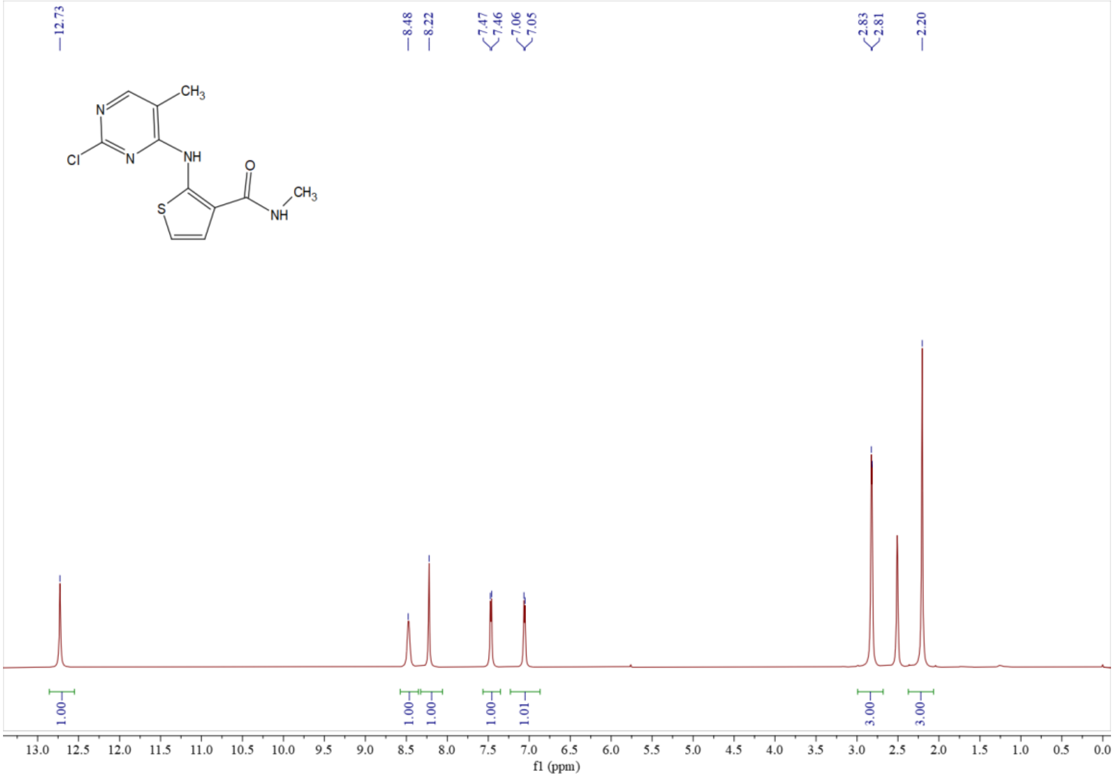

Supplementary Fig S3. ^{1}H NMR spectrum of compound **A1**

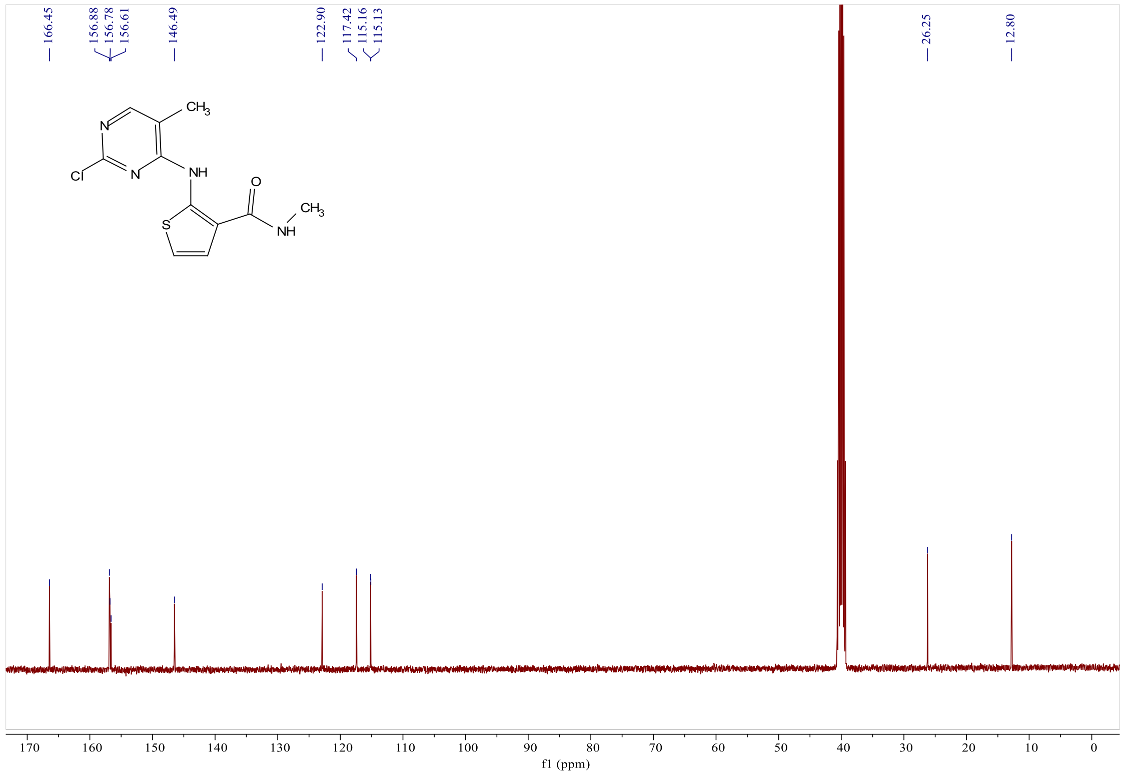

Supplementary Fig S4. ^{13}C NMR spectrum of compound **A1**

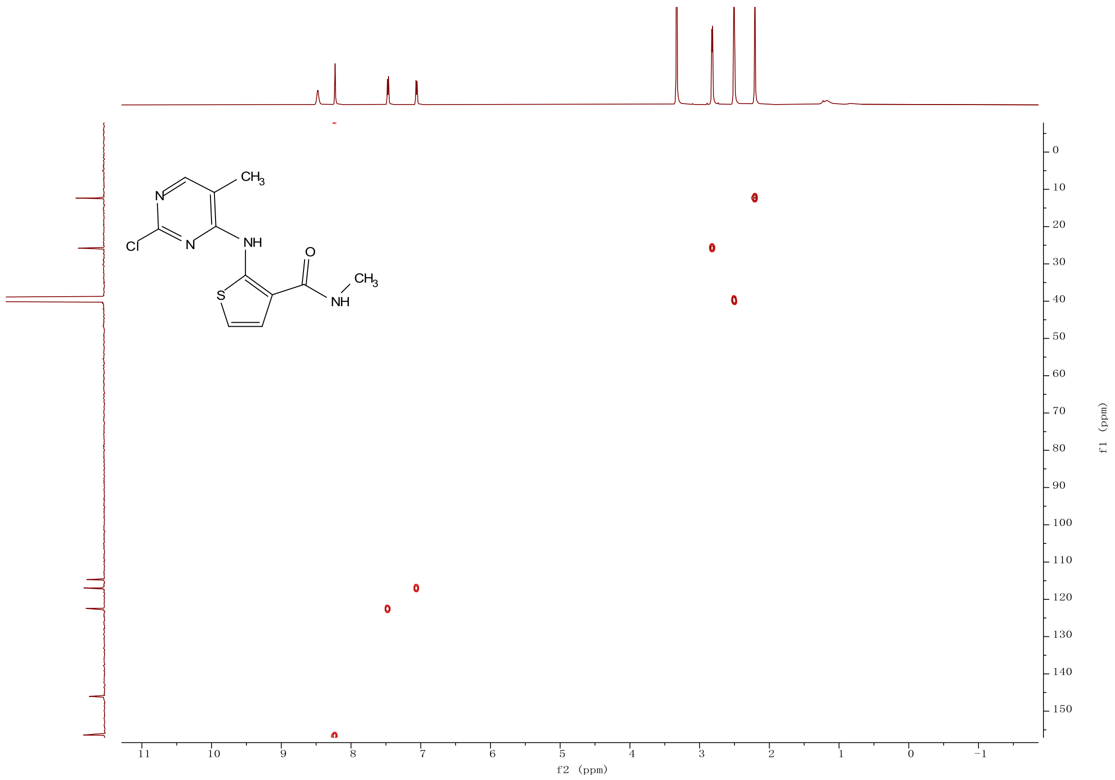

Supplementary Fig S5. HMQC spectrum of compound **A1**

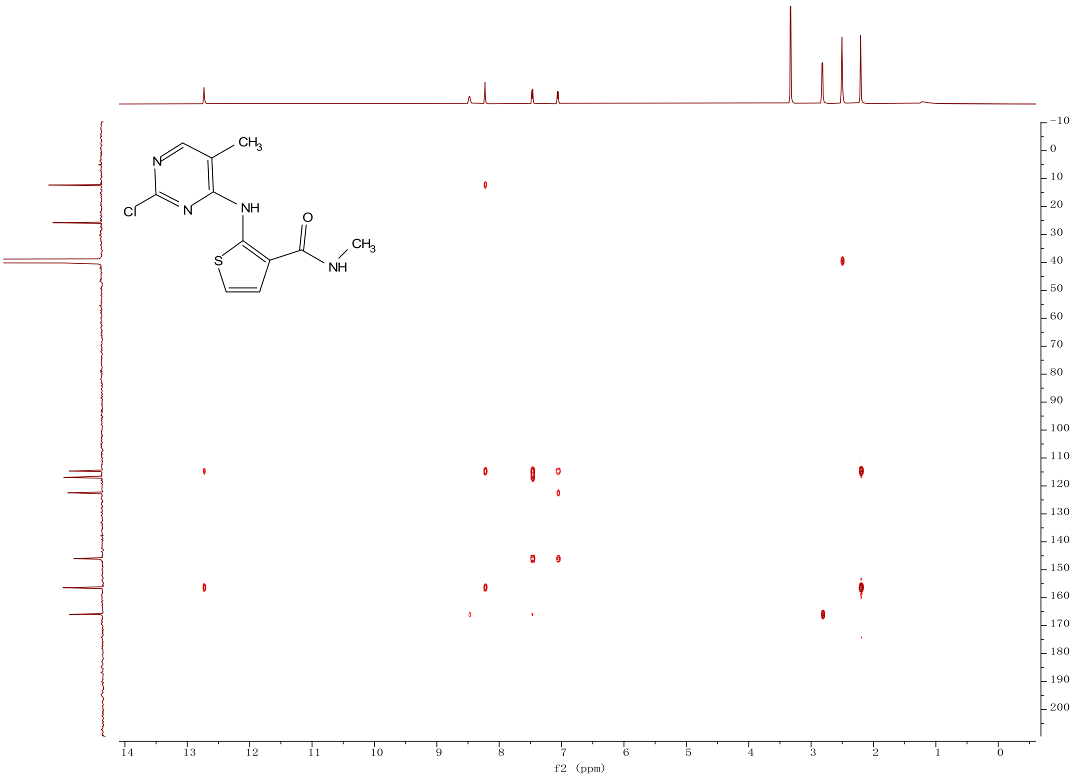

Supplementary Fig S6. HMBC spectrum of compound **A1**

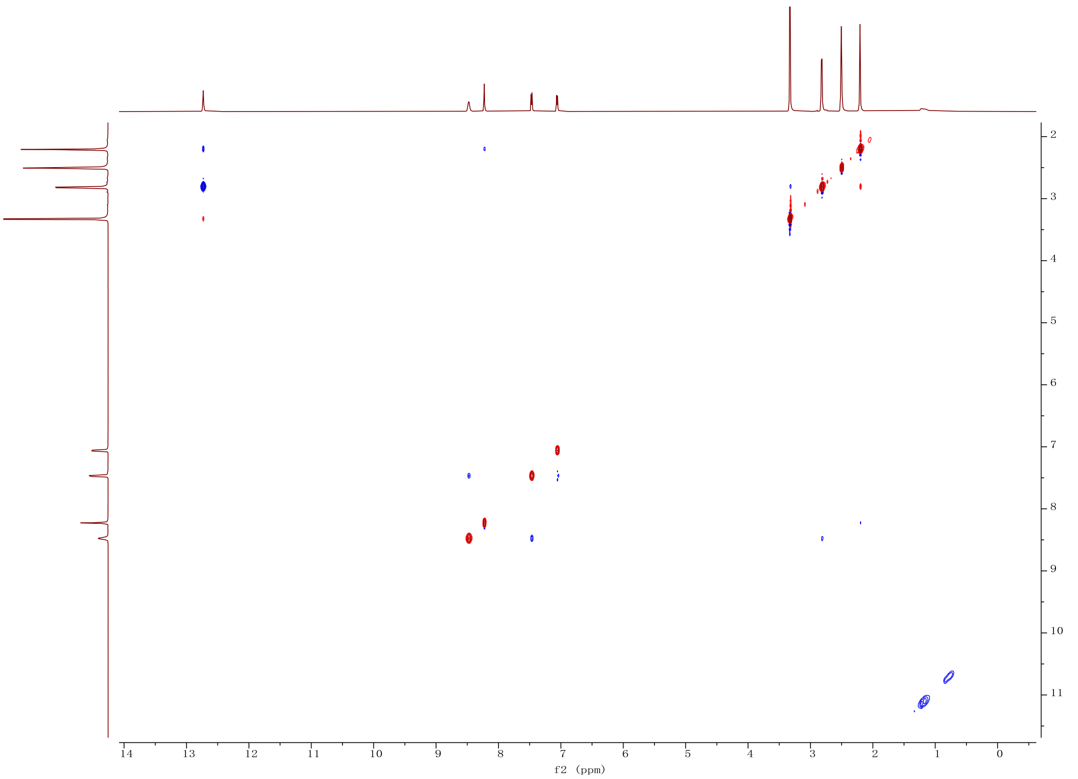

Supplementary Fig S7. NOESY spectrum of compound **A1**

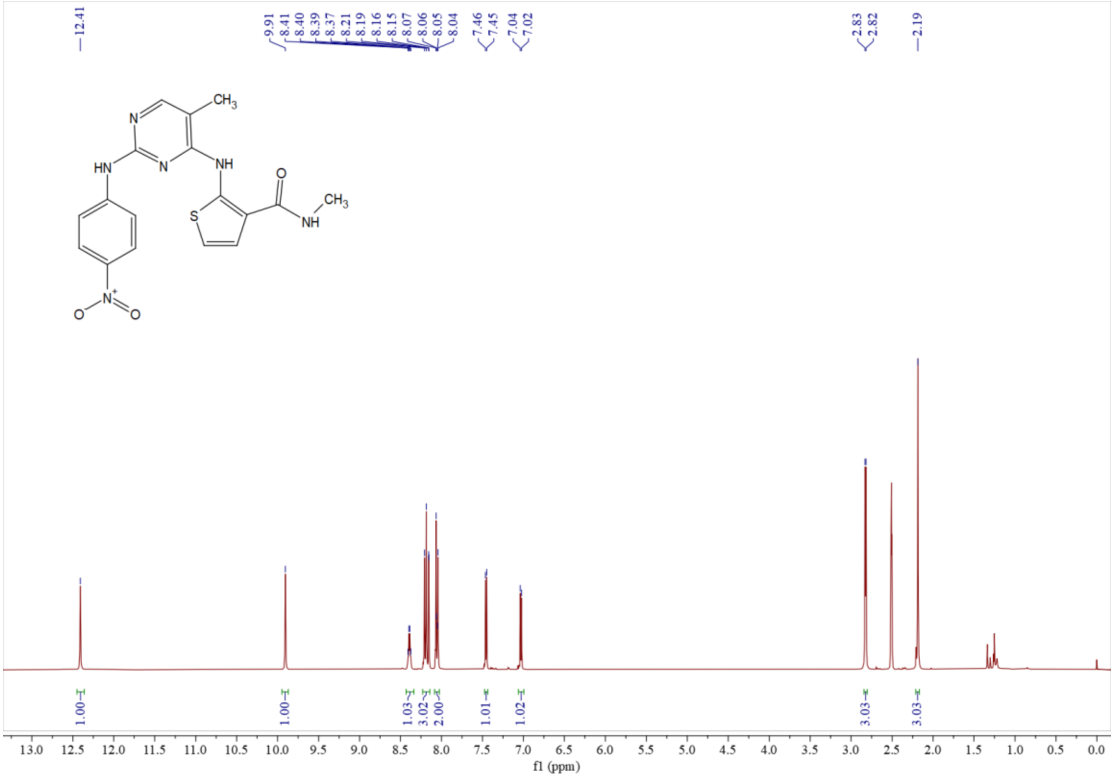

Supplementary Fig S8. ^{1}H NMR spectrum of compound **B1**

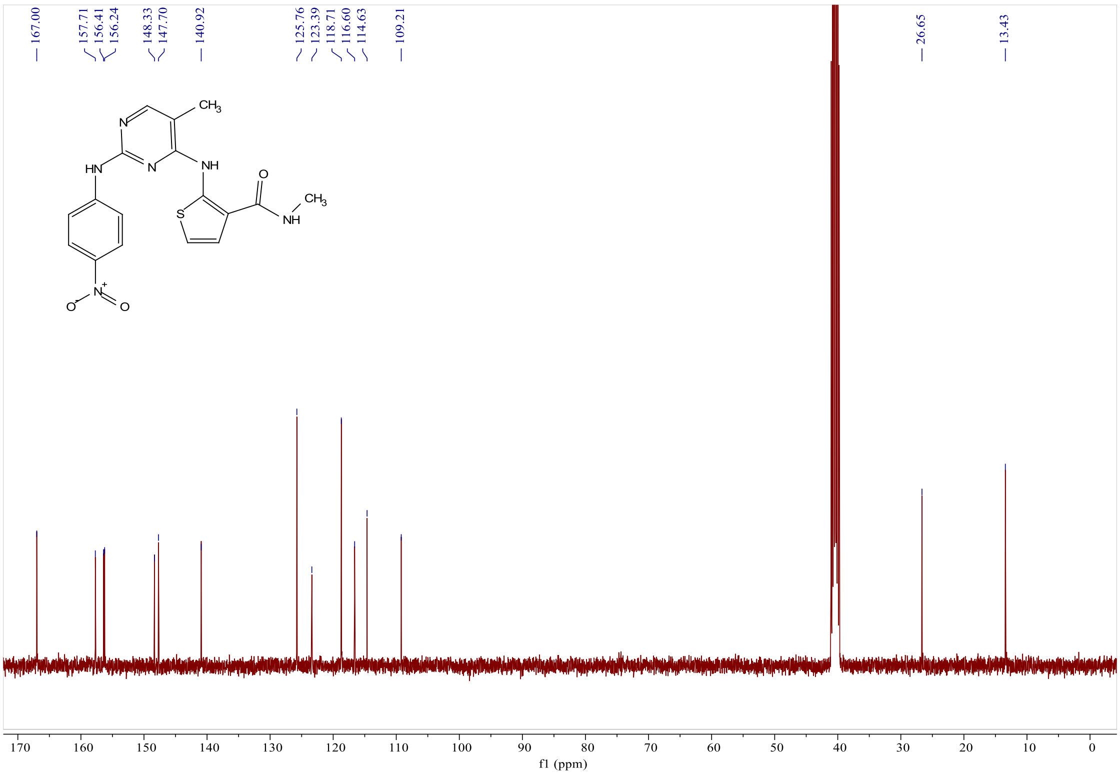

Supplementary Fig S9. ^{13}C NMR spectrum of compound **B1**

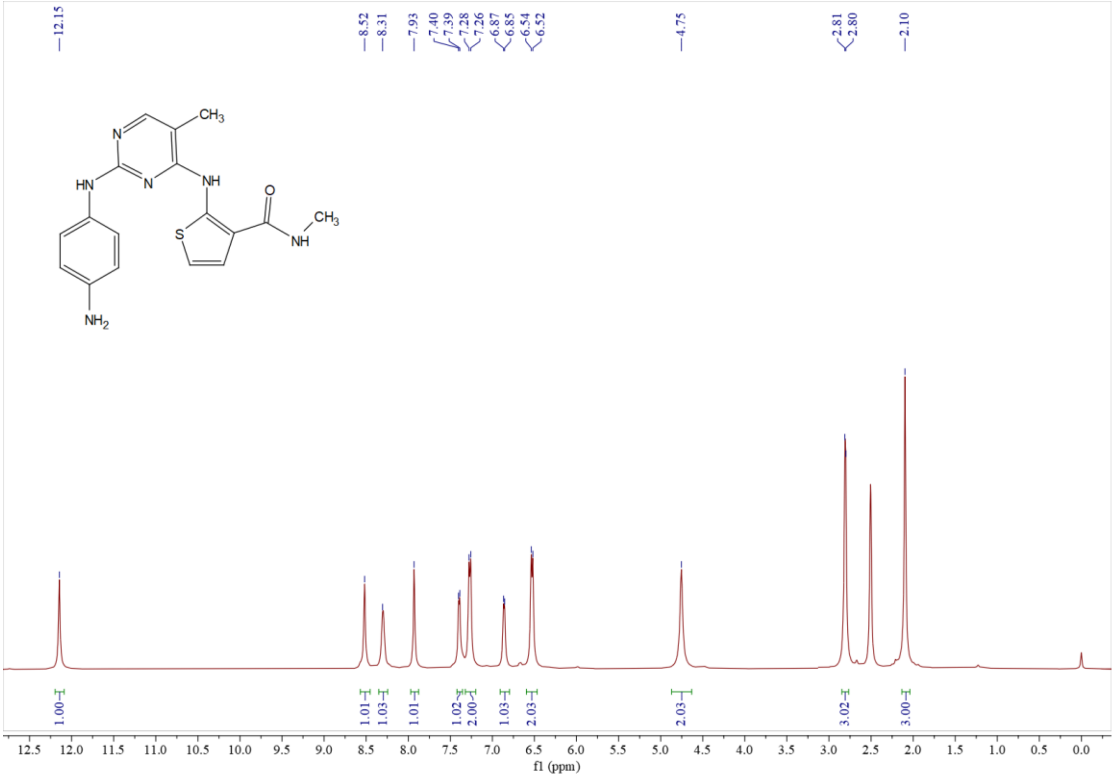

Supplementary Fig S10. ^{1}H NMR spectrum of compound **C1**

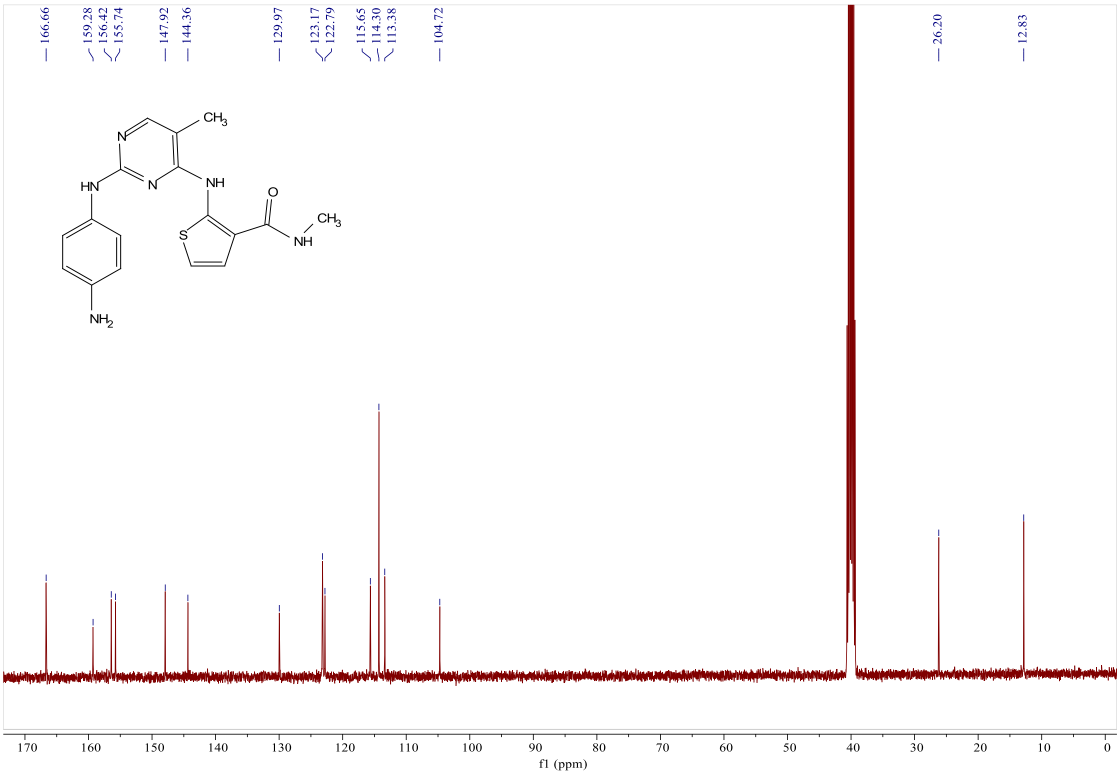

Supplementary Fig S11. ^{13}C NMR spectrum of compound **C1**

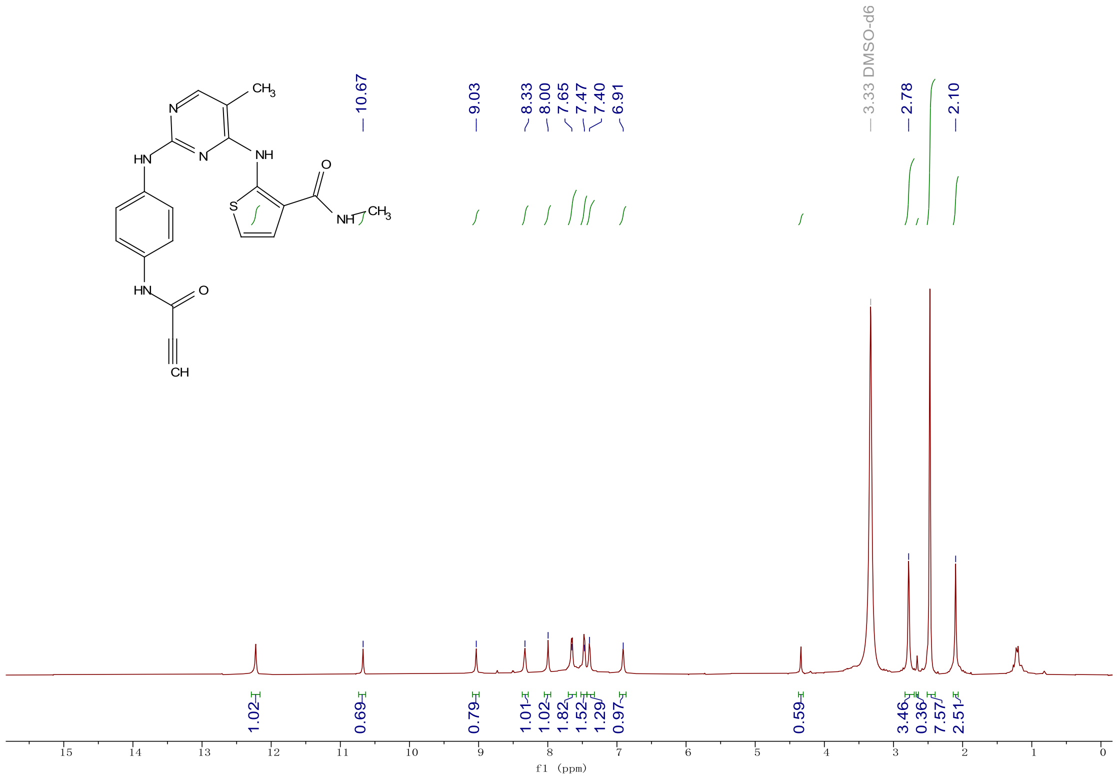

Supplementary Fig S12. ^{1}H NMR spectrum of compound **H-120**

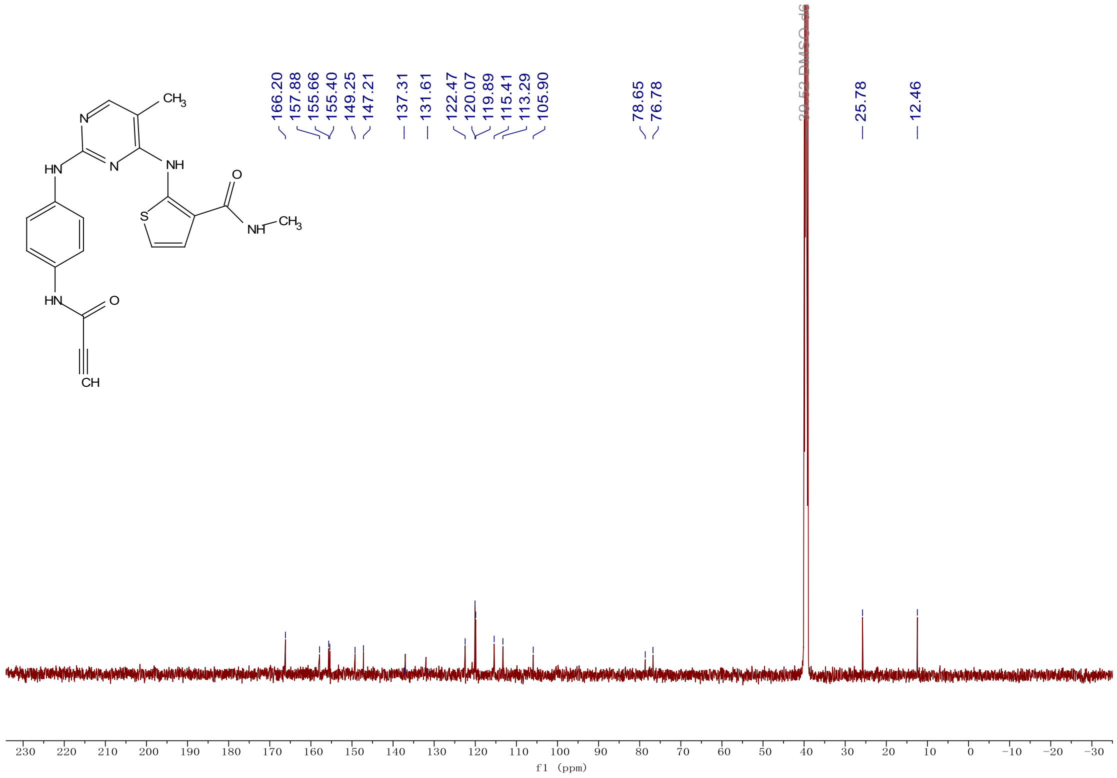

Supplementary Fig S13. ^{13}C NMR spectrum of compound **H-120**

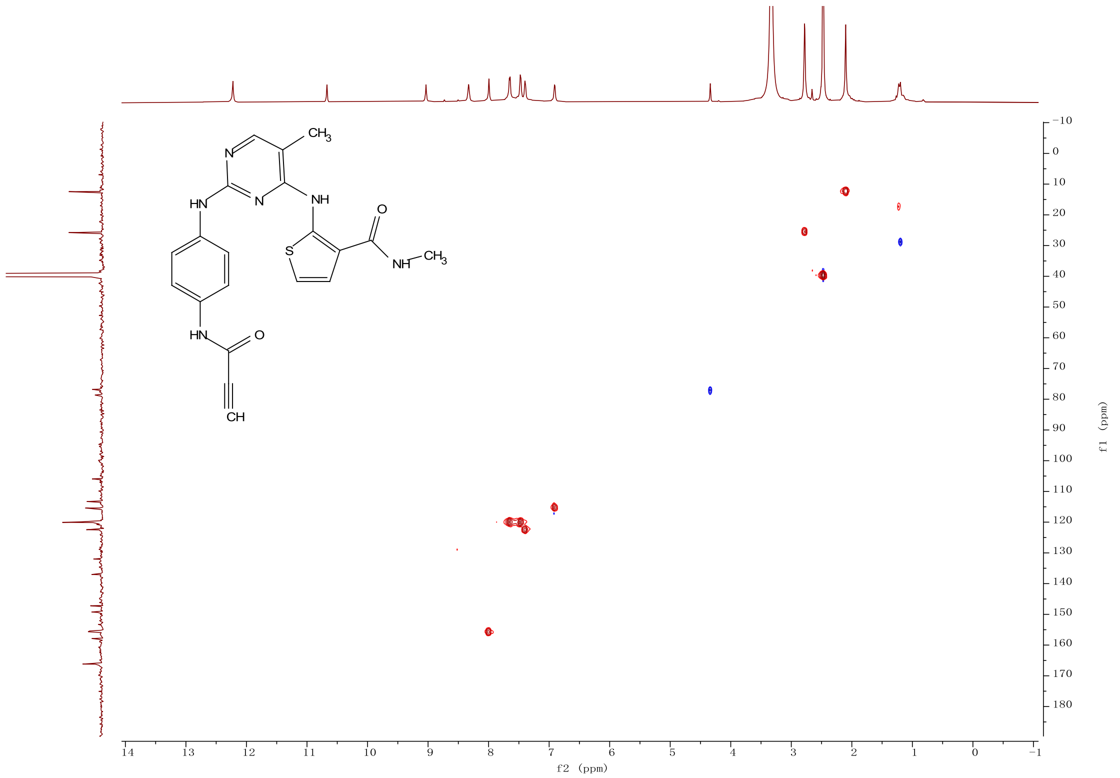

Supplementary Fig S14. HSQC spectrum of compound **H-120**

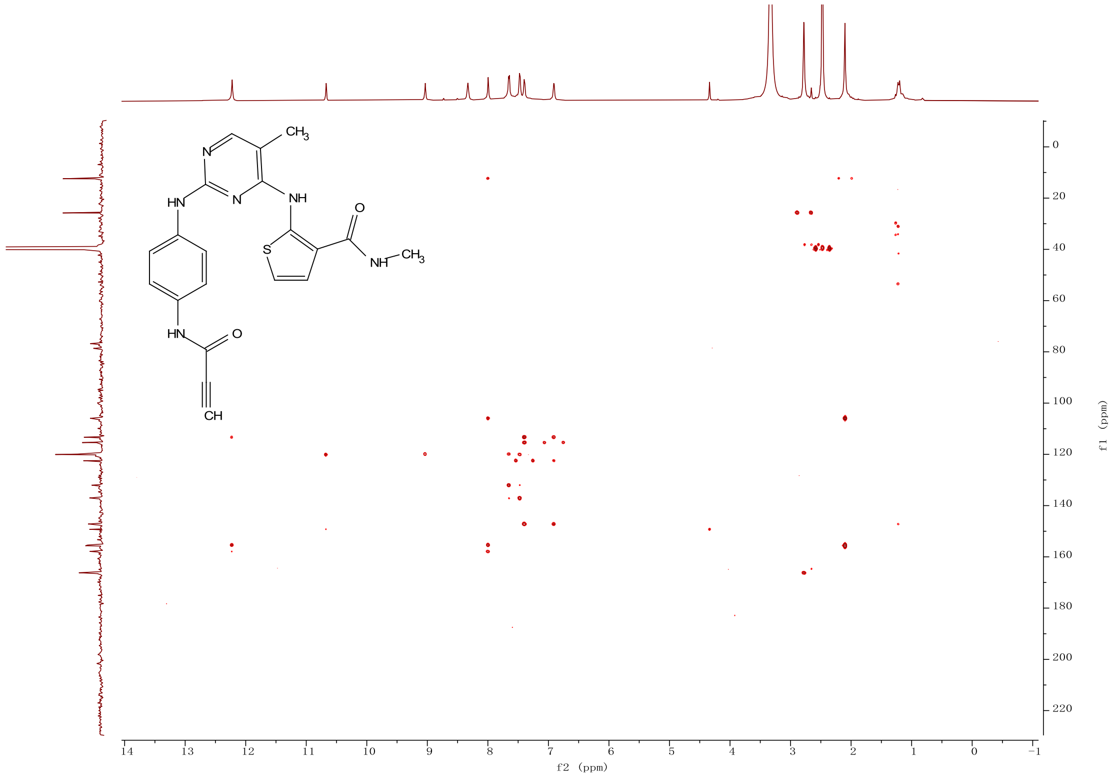

Supplementary Fig S15. HMBC spectrum of compound **H-120**

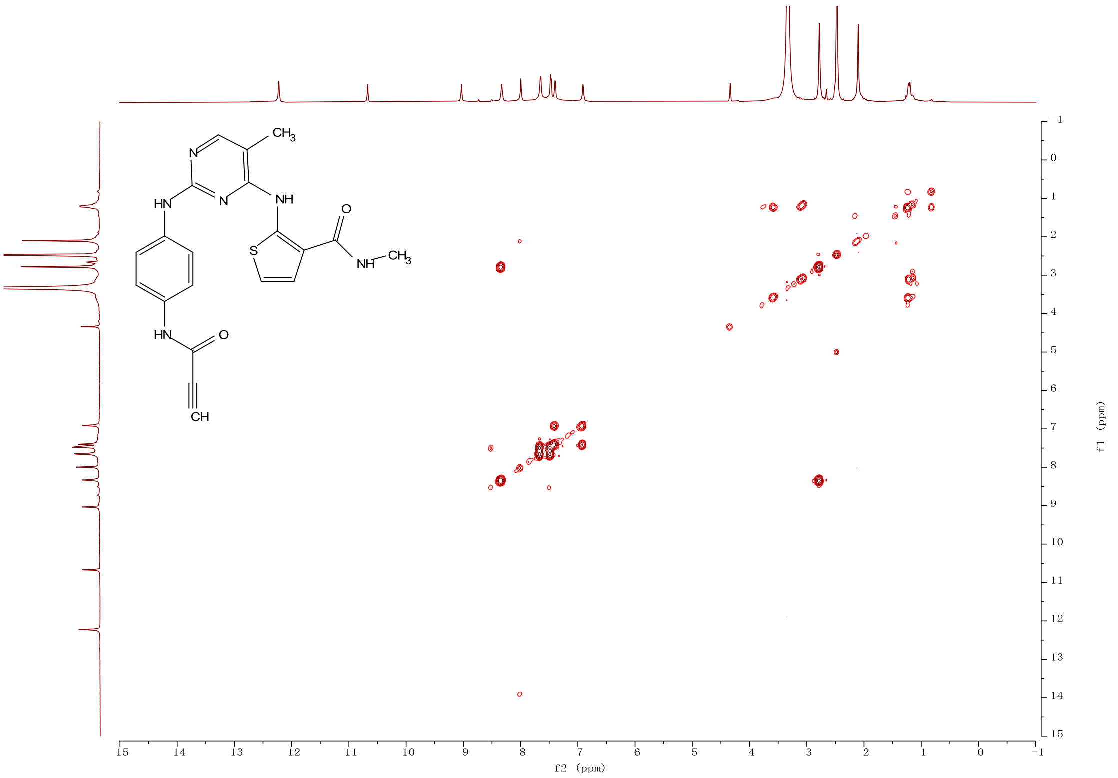

Supplementary Fig S16. ^{1}H-^{1}H COSY spectrum of compound **H-120**

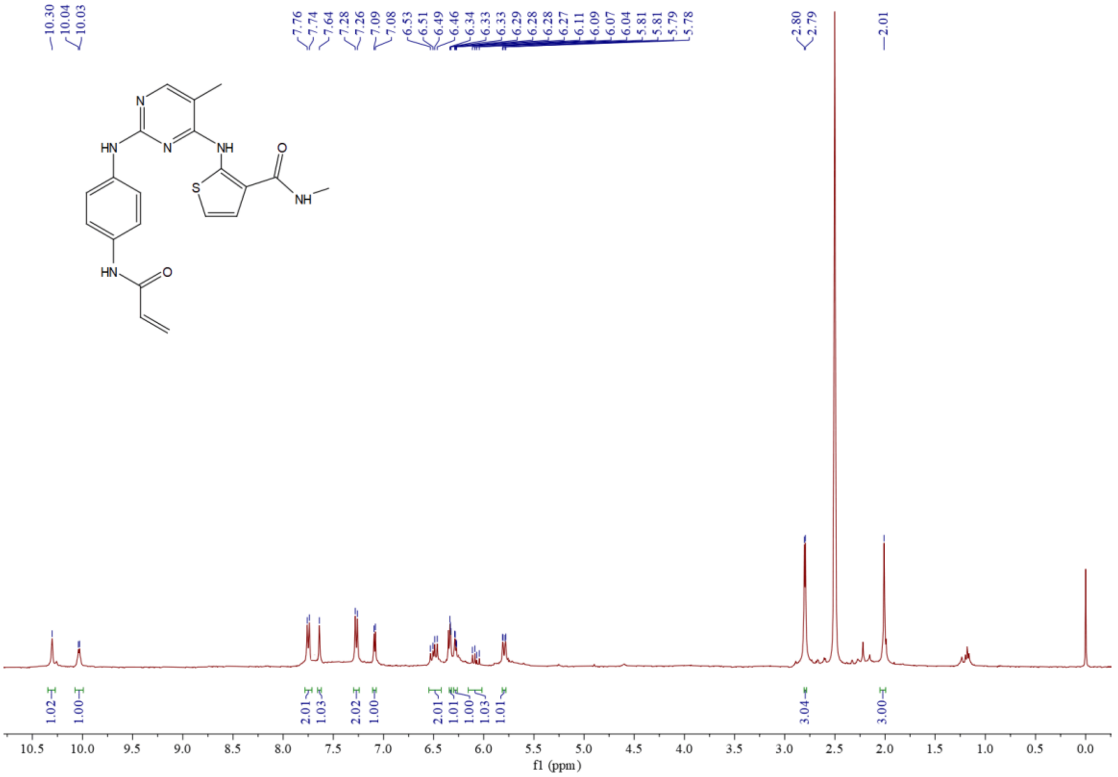

Supplementary Fig S17. ^{1}H NMR spectrum of compound **H-121**

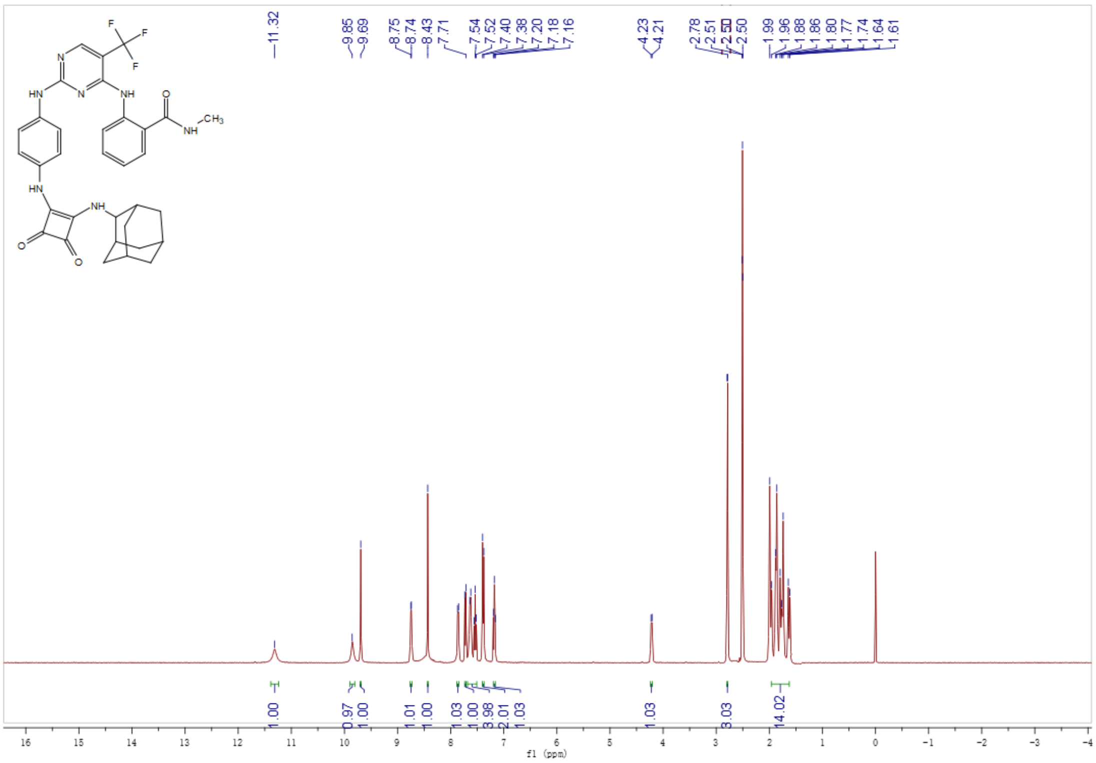

Supplementary Fig S18. ^{1}H NMR spectrum of compound **H-122**

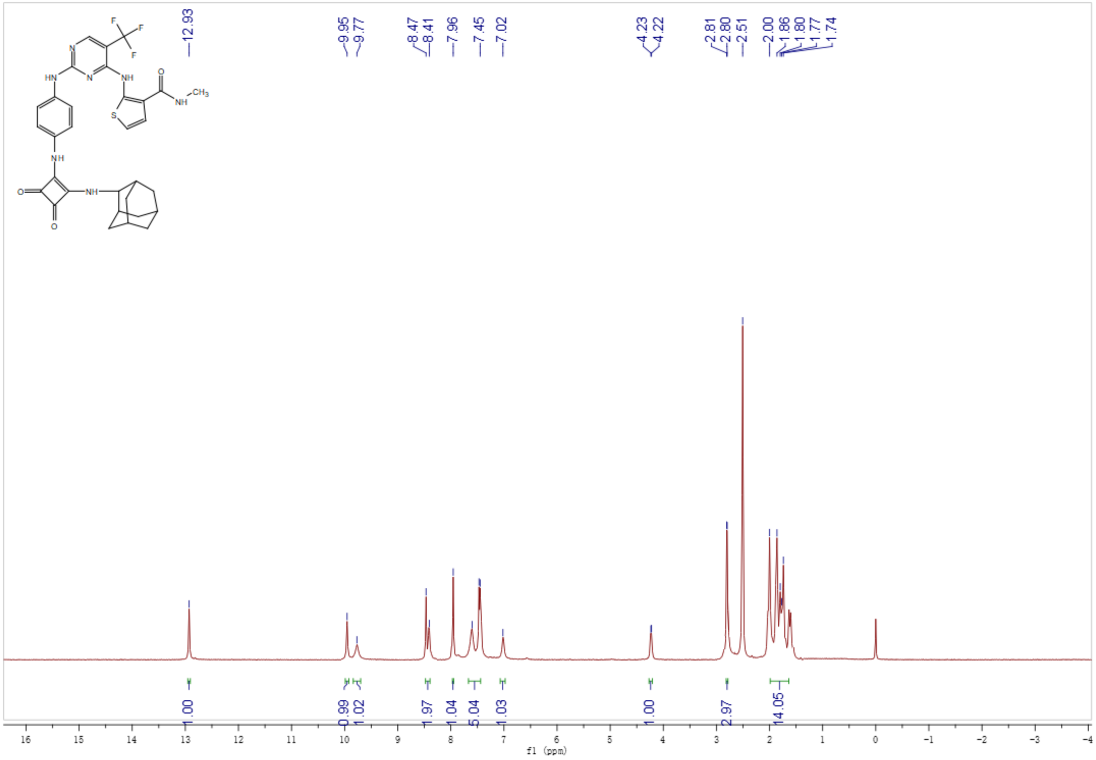

Supplementary Fig S19. ^{1}H NMR spectrum of compound **H-123**
